# Cycloolivil Isolated from *Nardostachys jatamansi* Inhibits TNF-α/IFN-γ-Induced Chemokine Production by Blocking NF-κB and JAK/STAT Activation in HaCaT Keratinocytes

**DOI:** 10.3390/ijms25063342

**Published:** 2024-03-15

**Authors:** Chi-Su Yoon, Hwan Lee, Zhiming Liu, Linsha Dong, Gyoyoung Lee, Nayeon Kim, Hyuncheol Oh, Dong-Sung Lee

**Affiliations:** 1 College of Pharmacy, Wonkwang University, Iksan 54538, Republic of Korea; ycs1991@naver.com; 2 Research Institute of Pharmaceutical Sciences, College of Pharmacy, Chosun University, Gwangju 61452, Republic of Korea; ghksdldi123@hanmail.net (H.L.); lzmqust@126.com (Z.L.); donglinsha011@163.com (L.D.); dlrydud612@naver.com (G.L.); rlaskdus1209@naver.com (N.K.)

**Keywords:** *Nardostachys jatamansi*, anti-skin inflammation, keratinocytes, JAK/STAT, NF-κB

## Abstract

*Nardostachys jatamansi* is widely used as a traditional medicine in Asian countries. Numerous recent studies have reported the biological activities of its secondary metabolites and extracts. In this study, a total of 14 components were isolated, including cycloolivil and 2-(3′-hydroxy-5′-ethoxyphenyl)-3-hydroxylmethyl-7-methoxy-2,3-dihydrobenzofuran-5-carboxylic acid, which were first discovered in *N. jatamansi*. The isolated compounds were investigated for their anti-inflammatory effects on HaCaT keratinocytes and their potential to alleviate skin inflammation. The results of the screening revealed that cycloolivil and 4β-hydroxy-8β-methoxy-10-methylene-2,9-dioxatricyclo[4.3.1.0^3,7^]decane reduced the production of inflammatory cytokines induced by TNF-α/IFN-γ, such as IL-6, IL-8, and RANTES, in keratinocytes. This study focused on exploring the biological effects of cycloolivil, and the results suggested that cycloolivil inhibits the expression of COX-2 proteins. Further mechanistic evaluations confirmed that the anti-inflammatory effects of cycloolivil were mediated by blockage of the NF-κB and JAK/STAT signaling pathways. These results suggest that cycloolivil isolated from *N. jatamansi* could be used to treat skin inflammatory diseases.

## 1. Introduction

Atopic dermatitis is an acute or chronic inflammatory skin disease characterized by persistent skin rashes. The main symptoms include itching, dry skin, and inflammation [1,2]. This disease primarily affects infants and young children, and its prevalence has been steadily increasing worldwide in recent years [3,4,5]. Therefore, the development of materials for early treatment will play a pivotal role in preventing and managing atopic dermatitis. The skin is composed of the epidermis, dermis, subcutaneous fat, and muscles. The epidermis, the outermost layer of the skin, is primarily composed of keratinocytes. Through the process of differentiation, keratinocytes play a crucial role in skin barrier formation and are important in inflammatory and immune responses upon exposure to stimuli [6]. However, when keratinocytes are excessively exposed to inflammatory cytokines and similar inflammatory inducers, the abnormal expression of cytokines and chemokines, including thymus and activation-regulated chemokine (TARC), is induced. This leads to the infiltration of inflammatory cells, such as white blood cells, into the inflamed areas [7]. Therefore, the exacerbation of skin inflammatory responses is known as a significant factor that induces atopic dermatitis.

Tumor necrosis factor (TNF)-α and interferon (IFN)-γ promote the production of inflammatory cytokines in human keratinocyte cell lines, such as HaCaT keratinocytes [8]. The overproduction of these inflammatory cytokines damages keratinocytes and reduces the rate of cell proliferation, contributing to the development of various inflammatory skin diseases, such as atopy and psoriasis [9]. When stimulated by TNF-α and IFN-γ, keratinocytes express inflammatory cytokines, chemokines, and intercellular adhesion molecule-1 (ICAM-1) [10]. These stimulations lead to the inappropriate activation of intracellular kinases, such as phosphoinositide 3-kinase (PI3K), Akt kinase, and janus kinase (JAK). This results in the activation of DNA binding by transcription factors, such as nuclear factor kappa-light-chain-enhancer of activated B cell (NF-κB) and signal transducer and activator of transcription (STAT) [11]. Therefore, the inhibition of NF-κB and JAK/STAT activation is a crucial molecular target for the treatment of skin inflammation.

The *Nardostachys* genus is a perennial herb that belongs to the Valerianaceae family. It grows in the high-altitude regions of the Himalayas and has been traditionally used in Korea and China owing to its medicinal properties, particularly in the treatment of depression, gastrointestinal disorders, headaches, and muscle pain [12]. Ayurveda, the traditional medical system in India, uses the rhizome of *Nardostachys jatamansi* as a tonic, antispasmodic, stimulant, and remedy for epilepsy. In Yūnānī medicine, a traditional medicine system of the Greek and Arabic cultural sphere, it has been used to treat indigestion, as well as liver and respiratory diseases [13]. In Korean traditional medicine, ‘Donguibogam’ recorded the use of *N. chinensis* for the treatment of liver spots on the skin [14]. Various studies have been conducted on plants belonging to the *Nardostachys* genus, and different components, including nardostachone, nardosinone, nardonoxide, kanshone A, and debilon, have been reported [15,16,17,18]. Additionally, previous studies have reported that *Nardostachys* exhibits various pharmacological effects, such as antioxidant, anti-inflammatory, neuroprotective, antiepileptic, cardioprotective, gastroprotective, antihypertensive, anxiolytic, and pigmentation effects [19,20]. Despite its historical use in skin treatment, research on the pharmacological activity concerning skin inflammation is limited. Therefore, this study aimed to isolate various compounds from *N. jatamansi* and investigate their anti-inflammatory effects on TNF-α/IFN-γ-induced HaCaT keratinocytes, with the goal of identifying potential therapeutic candidates for atopic dermatitis.

## 2. Results

### 2.1. Chemical Structures of Compounds Isolated from N. jatamansi

**Figure 1:**
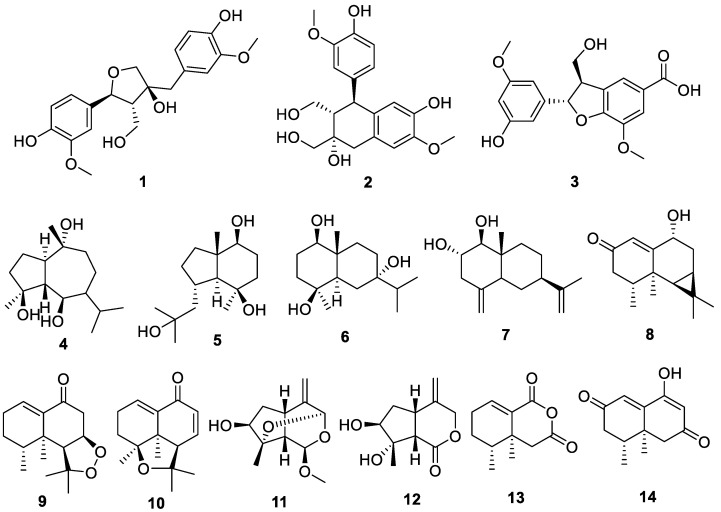
Chemical structures of the 14 compounds isolated from *Nardostachys jatamansi*.

Fourteen compounds were isolated from the ethyl acetate fraction partitioned from the methanol extracts of *N. jatamansi* through various chromatographic techniques, and their chemical structures were elucidated using nuclear magnetic resonance (NMR) analysis and mass spectrometry. The identified compounds included olivil (**1**), cycloolivil (**2**), 2-(3′-hydroxy-5′-ethoxyphenyl)-3-hydroxylmethyl-7-methoxy-2,3-dihydrobenzofuran-5-carboxylic acid (**3**), teuclatriol (**4**), bullatantriol (**5**), 1β,4β,7α-trihydroxyeudesmane (**6**), nardoeudesmol A (**7**), debilon (**8**), nardonoxide (**9**), nardosinone (**10**), 4β-hydroxy-8β-methoxy-10-methylene-2,9-dioxatricyclo[4.3.1.0^3,7^]decane (**11**), jatamanins A (**12**), 4α,5-dimethyl-1,3-dioxo1,2,3,4,4α,5,6,7-octahydronaphthalene (**13**), and kanshone M (**14**) (Figure 1). Notably, this is the first report of compounds **2** and **3** being isolated from *N. jatamansi*.

### 2.2. Inhibitory Effect of Compounds Isolated from N. jatamansi on Interleukin (IL)-6 Production in HaCaT Keratinocytes Induced by TNF-α/IFN-γ

**Figure 2:**
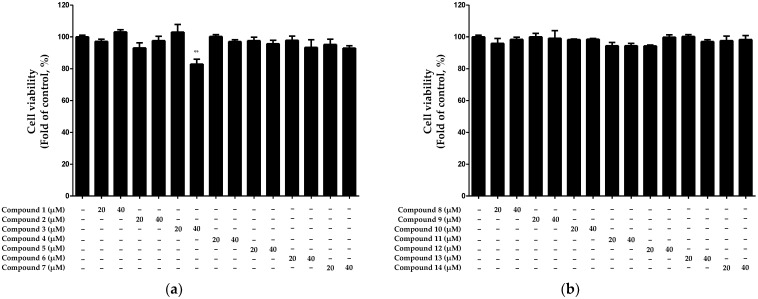
Cytotoxicity evaluation of compounds 1–7 (**a**) and 8–14 (**b**) isolated from *N. jatamansi*. Cells were individually treated with the 14 isolated compounds at concentrations of 20 and 40 μM for 24 h, and cytotoxicity was assessed using the MTT assay. Data are presented as the mean ± standard deviation (*n* = 3). ** *p* < 0.01, compared with the control group.

Cytotoxicity was evaluated using HaCaT keratinocytes to determine the optimal treatment concentrations for each of the 14 compounds isolated from *N. jatamansi*. The cytotoxicity evaluation was performed using the 3-(4,5-dimethylthiazol-2-yl)-2,5-diphenyltetrazolium bromide (MTT) assay, with treatments of each compound at concentrations of 20 and 40 μM. The results revealed that compound **3** exhibited significant cytotoxicity at a concentration of 40 μM, whereas the other compounds did not exhibit cytotoxicity up to a concentration of 40 μM (Figure 2).

**Figure 3:**
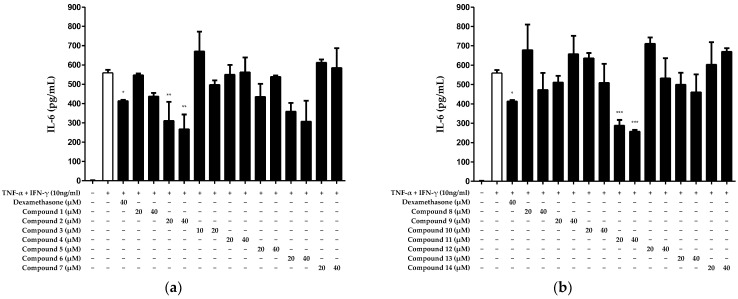
Inhibitory effects of compounds 1–7 (**a**) and 8–14 (**b**) isolated from *N. jatamansi* on TNF-α/IFN-γ-induced IL-6 production in HaCaT keratinocytes. Cells were treated with each of the 14 compounds at two concentrations (10 to 20 μM or 20 to 40 μM) and then induced with TNF-α/IFN-γ for 24 h. IL-6 levels were measured in cell supernatants. As a positive control group, dexamethasone was used for cell treatments at a concentration of 40 μM. Data are presented as the mean ± standard deviation (*n* = 3). * *p* < 0.05, ** *p* < 0.01, *** *p* < 0.001, compared with the TNF-α/IFN-γ treatment group.

Based on the results displayed in Figure 2, we investigated the inhibitory effects of the 14 compounds isolated from *N. jatamansi* on IL-6 production after treatment with non-toxic concentrations (10–40 μM). We treated HaCaT keratinocytes with each compound and subsequently activated the cells with the inflammatory cytokines TNF-α/IFN-γ. Next, we measured the IL-6 levels in the culture supernatants. The results suggested that compounds **2** and **11** significantly inhibited IL-6 production (Figure 3).

Given that only compounds **2** and **11** exhibited significant inhibitory effects on IL-6 production, we calculated their half-maximal inhibitory concentrations (IC_50_s) (Figure 3). The results revealed IC_50_ values of 31.05 ± 0.93 μM and 28.25 ± 0.21 μM for compounds **2** and **11**, respectively (Table 1).

### 2.3. Inhibitory Effect of Compounds Isolated from N. jatamansi on TNF-α/IFN-γ-Induced IL-8 and RANTES Production in HaCaT Keratinocytes

**Figure 4:**
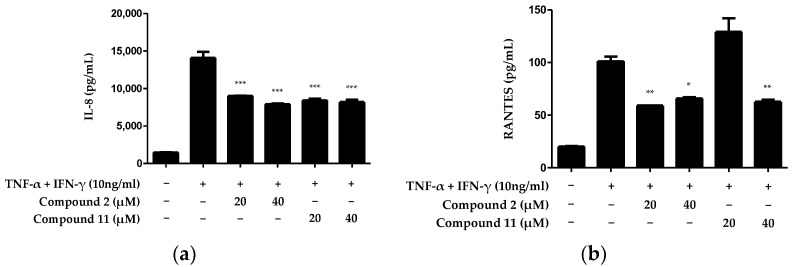
Inhibitory effects of compounds **2** and **11** on IL-8 (**a**) and RANTES (**b**) production in HaCaT keratinocytes induced by TNF-α/IFN-γ. IL-8 and RANTES were measured in cell supernatants after treatment with 20 and 40 μM concentrations of each compound and induction with TNF-α/IFN-γ for 24 h. Data are presented as the mean ± standard deviation (*n* = 3). * *p* < 0.05, ** *p* < 0.01, *** *p* < 0.001, compared with the TNF-α/IFN-γ-treated group.

We investigated the anti-inflammatory effects of the compounds that exhibited excellent IL-6-inhibitory effects on skin inflammation. Because of its complex structure, the skin performs various functions and plays a crucial role in protecting and maintaining the body from the external environment. The epidermis forms an immunological barrier that generates various cytokines and chemokines that can trigger inflammation [21]. This process is a crucial aspect of the skin’s ability to respond to external stimuli, as it mounts an immune response to defend the body. Therefore, we investigated the inhibitory effects of the two compounds on the extracellular release of the inflammatory cytokines IL-8 and regulated on activation, normal T cell expressed and secreted (RANTES). Compound **2** significantly inhibited the production of both IL-8 and RANTES at all tested concentrations. However, regarding compound **11**, while the inhibitory effect on IL-8 production was evident, the inhibition of RANTES production was not observed at a 20 μM concentration (Figure 4). Therefore, among the 14 compounds isolated from *N. jatamansi*, cycloolivil (**2**) exhibited the most potent inhibitory effect on inflammatory cytokines in TNF-α/IFN-γ-induced HaCaT keratinocytes.

### 2.4. Inhibitory Effect of Cycloolivil on TNF-α/IFN-γ-Induced Cyclooxygenase (COX)-2 Expression in HaCaT Keratinocytes

**Figure 5:**
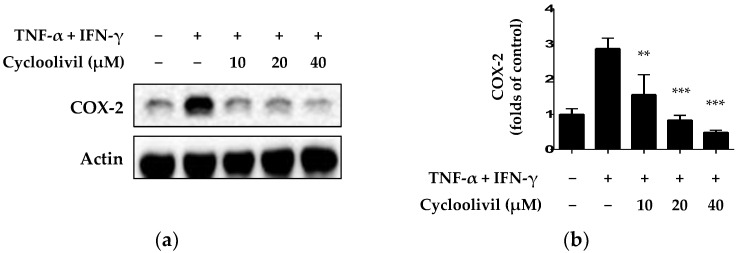
Inhibitory effect of cycloolivil on TNF-α/IFN-γ-induced expression of COX-2 in HaCaT keratinocytes. The results of the inhibitory effect on COX-2 were detected using western blot (**a**) and analyzed by ImageJ (**b**). Cells were pre-treated with cycloolivil at concentrations of 10–40 μM for 3 h, followed by stimulation with TNF-α/IFN-γ for 24 h. Data are presented as the mean ± standard deviation (*n* = 3). ** *p* < 0.01, *** *p* < 0.001, compared with the TNF-α/IFN-γ-treated group.

Under inflammatory conditions, keratinocytes express inflammatory proteins, such as COX-2. Specifically, an isoform of COX, the COX-2 enzyme, plays an important role in allergic reactions and inflammation [22]. Therefore, we investigated the inhibitory effect of cycloolivil on expression of the inflammatory protein COX-2 in HaCaT keratinocytes exposed to TNF-α/IFN-γ. The results demonstrated the significant inhibition of COX-2 expression (Figure 5).

### 2.5. Effect of Cycloolivil on Regulation of the JAK/STAT Signaling Pathway in HaCaT Keratinocytes

**Figure 6:**
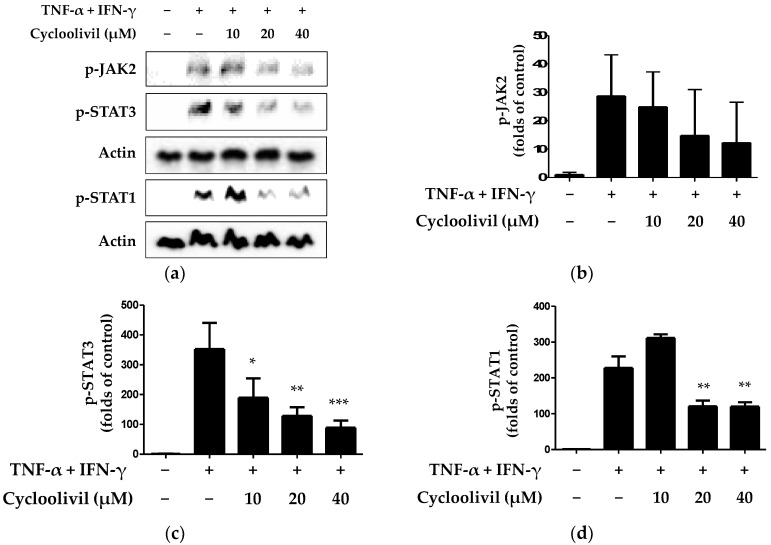
Effects of cycloolivil on the p- JAK2, p-STAT3, and p-STAT1 signaling pathways in HaCaT keratinocytes (**a**–**d**). Cells were pre-treated with the indicated concentrations of cycloolivil for 3 h and then stimulated with TNF-α/IFN-γ for 15 min. Protein expression was measured using western blotting. Data are presented as the mean ± standard deviation (*n* = 3). * *p* < 0.05, ** *p* < 0.01, *** *p* < 0.001, compared with the TNF-α/IFN-γ-treated group.

The stimulation of keratinocytes with TNF-α/IFN-γ induces the release of cytokines and chemokines through various signaling pathways. The activation of keratinocytes by inflammatory cytokines involves signaling through the JAK/STAT cascade [23]. Additionally, this pathway regulates immune responses induced by IFN-γ and regulates a variety of important biological responses, including immune functions. JAKs and STAT, which are activated by stimuli, transmit signals from cell surface receptors, and activated STAT1 specifically induces the expression of various inflammatory genes [24]. Based on this theory, we investigated whether cycloolivil is involved in JAK/STAT pathway activation in HaCaT keratinocytes induced by TNF-α/IFN-γ. Our findings demonstrated that cycloolivil significantly inhibited the activation of p-STAT3 and p-STAT1 but did not affect the activation of p-JAK2 (Figure 6).

### 2.6. Effect of Cycloolivil on Regulation of the NF-κB Signaling Pathway in HaCaT Keratinocytes

**Figure 7:**
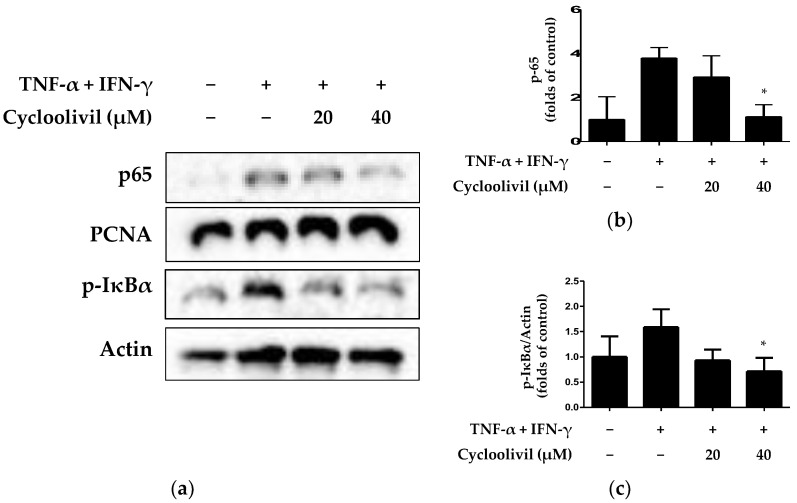
Effects of cycloolivil on the NF-κB signaling pathway (**a**–**c**) in HaCaT keratinocytes. Cells were pre-treated with the indicated concentrations of cycloolivil for 3 h and then stimulated with TNF-α/IFN-γ(+) for 15 min. The levels of p65 and p- IκBα in fractions obtained through cytoplasmic and nuclear isolation were determined using western blotting. Data are presented as the mean ± standard deviation (*n* = 3). * *p* < 0.05, compared with the TNF-α/IFN-γ-treated group.

The NF-κB signaling pathway activates various inflammatory genes within HaCaT keratinocytes undergoing an inflammatory response. NF-κB activation is involved in the induction of inflammatory cytokines and proteins, such as IL-6, IL-8, and COX-2 [25]. Therefore, the appropriate regulation of NF-κB prevents tissue damage while maintaining normal immune and inflammatory responses. Consequently, the modulation of NF-κB is primarily utilized in mechanistic studies related to inflammatory diseases and immune functions. Accordingly, we investigated whether cycloolivil is involved in activation of the NF-κB pathway in HaCaT keratinocytes induced by TNF-α/IFN-γ. The results revealed that cycloolivil significantly inhibited the activation of p65 and p-inhibitor of κB (IkBa) (Figure 7).

## 3. Discussion

The *Nardostachys* genus contains various types of terpene skeletons, such as mono-, sesqui-, di-, and tri-terpenes. Moreover, it comprises a diverse array of lignans. Our previous high-pressure liquid chromatography (HPLC) profiling of 20% ethanolic extracts of *N. jatamansi* revealed the presence of narchinol-type sesquiterpenes and pinoresinol-type lignans. In particular, narchinol-type compounds have strong anti-neuroinflammatory effects in microglial cell lines [26], which correlate with the remarkable effects demonstrated by *Nardostachys* extracts [27]. Our ongoing exploration of bioactive compounds from natural products prompted the screening of the anti-inflammatory effects of 14 compounds isolated from *N. jatamansi*. The findings revealed that only compounds **2** and **11** exhibited inhibitory effects against the production of the cytokine IL-6 in HaCaT cells induced by TNF-α/IFN-γ (Figure 3).

The epidermis is the outer layer of the skin and is mainly composed of keratinocytes. Keratinocytes play an indispensable role as inherent constituents of the skin barrier, acting as a physical defense against environmental threats. Keratinocytes also play an active protective role against pathogenic invasion. This capability is particularly important when the skin’s physical defenses are compromised due to injury. During the inflammatory phase of the healing process, keratinocytes act as immunomodulators, regulating inflammation through a rigorously coordinated network of inflammatory cascades. This system is activated through keratinocyte-receptor communication with the surroundings in a paracrine- and autocrine-dependent manner [28]. Keratinocytes can express the receptors of TNF-α and IFN-γ. Stimulation with these cytokines induces the expression of various pro-inflammatory genes in keratinocytes, including CXCL5, CXCL8 (also known as IL-8), and ICAM-1 [9]. Based on this theory, we confirmed the inhibitory effects of compounds **2** and **11** on IL-8 and RANTES production in HaCaT cells induced by TNF-α/IFN-γ. Both compounds inhibited the production of IL-8 and RANTES. However, no inhibitory effect on RANTES production was observed at 20 μM for compound **11** (Figure 4).

We conducted a literature search to gather information on the known biological activities of compounds **2** and **11**. Based on HR-ESIMS and NMR analyses, the molecular formula of compound **2** was determined to be C_20_H_24_O_7_, with nine unsaturations. The ^1^H- and ^13^C-NMR data suggested the presence of two benzene rings, two methoxy group, two oxygenated-methine and -methylene groups, a methine, and a methylene group. The MS and NMR pattern was similar to that observed for compound **1**; based on a comparison of NMR data from reported literature, compound **1** was identified as olivil, and compound **2** as cycloolivil. The spectroscopic data of compound **3** revealed a lignan-type chemical structure with carboxylic acid, which differs from compounds **1** and **2**, and as a result, compound **3** was identified as 2-(3′-hydroxy-5′-ethoxyphenyl)-3-hydroxylmethyl-7-methoxy-2,3-dihydrobenzofuran-5-carboxylic acid. In addition, HR-ESIMS and NMR data of compound **11** suggested that its molecular formula is C_11_H_16_O_4_, with four unsaturations. The ^1^H- and ^13^C-NMR data suggested the presence of an allyl group, three oxygenated-methines, a methoxy, two methines, a methylene, and a methyl group. Interestingly, HMBC correlation showed an oxygenated bridge connection between carbon 1 to 2 in the iridoid-skeleton, and from the comparison of NMR data from the reported literature, the chemical structure was determined as 4β-hydroxy-8β-methoxy-10-methylene-2,9-dioxatricyclo[4.3.1.0^3,7^]decane. We further investigated the purity of the bioactive compounds 2 and 11 via HPLC analysis; as a result, the purity of 2 was 93% and that of 11 was 99% (Figures S31 and S32). These compounds have exhibited inhibitory effects on inflammatory cytokines and chemokines. Compound **2** has demonstrated a range of biological activities, including anti-aggregant, antiproliferative, hepatoprotective, antibacterial, and antioxidant effects [29,30,31,32,33]. Similarly, compound **11** is known to have neuroprotective and anti-neuroinflammatory properties [34,35]. Considering the inhibitory effect of compound 2 (cycloolivil) on the production of IL-6, IL-8, and RANTES and its known biological activities, we performed further studies on its anti-inflammatory effects, which have not been previously investigated. Before examining the inflammatory signaling pathway, we investigated the expression of COX-2, an important molecules associated with inflammation. COX-2 is an enzyme involved in generating compounds associated with inflammation, and its expression tends to increase during inflammation. Considering this role, we explored whether cycloolivil influenced the expression of COX-2. The results confirmed that cycloolivil significantly regulated the expression of COX-2 (Figure 5). These findings predicted that cycloolivil inhibits skin inflammation through the regulation of COX-2 expression.

The JAK/STAT signaling pathway is widely expressed in various cells and plays an important role in many immunological and inflammatory diseases [9]. The activation of the JAK/STAT signaling pathway in keratinocytes is triggered by various stimuli, including TNF-α/IFN-γ stimulation [23]. The activation of this pathway plays an important role in the signaling of cytokines, chemokines, and growth hormones. When a ligand binds to the cell surface-bound JAK receptor, tyrosine is phosphorylated and forms a docking site in the cytoplasmic tail of the receptor. This docking site allows for the binding of STATs, which are subsequently phosphorylated and activated. When the same or different types of STATs are formed, phosphorylated STATs move into the nucleus and promote gene transcription [24]. Our study focused specifically on the JAK/STAT pathway and confirmed that this pathway could be targeted for the treatment of skin irritation. The results demonstrated that cycloolivil inhibited JAK2, STAT1, and STAT3 phosphorylation in keratinocytes, suggesting a role in mitigating skin inflammation (Figure 6).

Inflammatory responses primarily enhance NF-κB, inducing the transcription of downstream target genes, such as IL-1, IL-6, and TNF-α. This response also involves chemokines, such as IL-8 and the inflammatory enzyme COX-2 [25]. Therefore, the regulation of NF-κB activation plays a crucial role in modulating inflammatory responses. NF-κB activation primarily occurs through the nuclear translocation of p65, facilitated by the phosphorylation of inhibitor of κB (IκB) proteins. Translocated p65 in the nucleus, in turn, promotes IκB transcription, regulating the inflammatory response. Our experimental results confirmed that cycloolivil significantly inhibits the nuclear translocation and phosphorylation of IκBα in keratinocytes exposed to TNF-α/IFN-γ. These findings suggest that cycloolivil may effectively regulate the inflammatory signaling pathway, contributing to the suppression of the inflammatory response (Figure 7). Thus, the results of this study suggest the potential efficacy of cyclooilivil in treating inflammatory conditions, highlighting the necessity for further research, including experimental in vivo studies, to deepen our understanding.

## 4. Materials and Methods

### 4.1. Materials, Reagents, and Instruments

Reagent-grade solvents were used for extraction and column chromatography (CC). For CC, YMC ODS-A (C18) (YMC, Kyoto, Japan), Cytiva Sephadex™ LH-20 (Cytiva, Marlborough, MA, USA), and silica gel (Merck, Darmstadt, Germany) were used as the resin. Subsequently, 1D and 2D NMR spectra were acquired in chloroform-*d*, acetone-*d*_6_, and dimethyl sulfoxide (DMSO)-*d*_6_ (Cambridge Isotope Laboratories, Inc., Andover, MA USA) using a JEOL JNM ECP-400 spectrometer (400 MHz ^1^H and 100 MHz ^13^C; JEOL Ltd., Akishima, Japan). HRESI-MS data were obtained using an AB Sciex Triple TOF 4600 instrument (AB Sciex Pte. Ltd., Framingham, MA, USA). For HPLC purification, the YL-9100 system (YoungLin, Anyang, Republic of Korea) was used, and HPLC separations were performed on a prep-C18 column (21.2 × 150 mm; 5 μm particle size) with a flow rate of 5 mL/min and a semiprep-C18 column (10 × 250 mm; 5 μm particle size) with a flow rate of 3 mL/min. The plates used for cell experiments were supplied by Corning (Amsterdam, The Netherlands), and all cell media were supplied by ATCC (Manassas, VA, USA). Human TNF-α, IFN-γ, and enzyme-linked immunosorbent assay (ELISA) kits for IL-8, IL-6, and RANTES were purchased from Biolegend (San Diego, CA, USA). The actin, proliferating cell nuclear antigen, and horseradish peroxidase-conjugated anti-mouse and anti-rabbit IgG antibodies were purchased from Santa Cruz Biotechnology (Dallas, TX, USA). p-IκBα, IκBα, p65, p-JAK2, p-STAT1, and p-STAT3 were purchased from Cell Signaling Technologies (Danvers, MA, USA). All reagents not mentioned in this study, including dexamethasone, which was used as a positive control, were purchased from Sigma-Aldrich (St. Louis, MO, USA).

### 4.2. Extraction, Isolation, and Structural Identification

*N. jatamansi* was purchased from a standard commercial source (KwangMyungDang, Ulsan, Republic of Korea), and its identity was confirmed by the Korean Drug Test Laboratory (Seoul, Republic of Korea). Voucher specimens (WK-2016-03) were deposited in the College of Pharmacy Herbarium at Wonkwang University. *N. jatamansi* (5 kg) was extracted in MeOH (24 L) via sonication for 2 h to obtain a MeOH extract (663 g). The MeOH extract was dissolved in a mixture of H_2_O (8 L) and MEOH (1 L), followed by partitioning with n-hexane, chloroform, EtOAc, and *n*-butanol (18 L each). The EtOAc fraction, NJ(E) (21.8 g), was subjected to silica gel CC and eluted with hexane:EtOAc (3.5:1–1:0) and EtOAc:MeOH (99:1–20:80) to yield 13 subfractions: (E)-S1–S13. Subfraction NJ(E)-S1 was subjected to normal phase-HPLC and eluted with hexane in EtOH gradient methods at 99–98% (0–20 min) to yield compounds **9** (t_R_ = 11.4 min, 11 mg) and **10** (t_R_ = 14.6 min, 2 mg). Subfraction NJ(E)-S3 was subjected to silica gel CC and eluted with hexane:EtOAc (3.5:1–0:1) to yield 11 subfractions, NJ(E)-S3-1–11. Subfractions NJ(E)-S3-1,2, and 3 were combined as NJ(E)-S3-A and subjected to C_18_ CC, eluting with MeOH in H_2_O (20–90%) to yield 13 subfractions, NJ(E)-S3A-1–13. Subfraction NJ(E)-S3A-10 was subjected to prep reversed phase (RP)-HPLC and eluted using MeOH in H_2_O (0.1 % formic acid) gradient methods at 35–85% (0–30 min) to yield compound **7** (t_R_ = 22.5, 3.4 mg). Subfraction NJ(E)-S3-6 was subjected to prep RP-medium pressure liquid chromatography and eluted with MeOH in H_2_O (40–100%) to yield compound **13** (125 mg). Subfractions NJ(E)-S4 and 5 were combined as NJ(E)-S(45) and subjected to C_18_ CC and elution with MeOH in H_2_O (10–100%) to yield 15 subfractions, NJ(E)-S(45)-1–15. Subfraction NJ(E)-S(45)-5 was subjected to silica gel CC and eluted with hexane:EtOAc (2:1–0:1) to yield eight subfractions, NJ(E)-S(45)-5-1~11. Subfraction NJ(E)-S(45)-5-8 was subjected to silica gel CC and eluted with chloroform:MeOH (15:1–1:1) to yield six subfractions: NJ(E)-S(45)-5-8-1–6. Subfraction NJ(E)-S(45)-5-8-3 was subjected to prep RP-HPLC and eluted using MeOH in H_2_O (0.1% formic acid) gradient methods at 30–50% (0–30 min) to yield compound **2** (t_R_ = 14.5 min, 5.3 mg). NJ(E)-S(45)-7 was subjected to silica gel CC and eluted with CH_2_Cl_2_:MeOH (9:1–2:1), yielding compound **1** (32 mg). Subfraction NJ(E)-S(45)-9 was subjected to C_18_ CC and eluted with MeOH in H_2_O (40–60%) to yield four subfractions, NJ(E)-S(45)-9-1–4. Subfraction NJ(E)-S(45)-9-2 was subjected to silica gel CC and eluted with chloroform:EtOAc (1.5:1–0:1) to yield seven subfractions: NJ(E)-S(45)-9-2-1~7. Subfraction NJ(E)-S(45)-9-2-6 was subjected to silica gel CC and eluted with hexane:acetone (6:4–0:1) to yield compound **3** (5.3 mg). The purification step and spectrometry data for compounds **4**, **5**, **6**, **8**, **12**, and **14** were described in our previous study [33,36]. The spectrometric data (HRESI-MS and NMR data) for compounds **1**, **2**, **3**, **7**, **9**, **10**, **11**, and **13** are available in the Supplementary Materials (Figures S1–S30).

### 4.3. Cell Culture

HaCaT keratinocytes were obtained from the ATCC (Manassas, VA, USA). Dulbecco’s modified Eagle’s medium (DMEM) (Gibco, Grand Island, NY, USA) containing 10% fetal bovine serum and 1% antibiotic–antimycotic solution (100 U/mL) was used as the cell medium. Cells were cultured in an incubator maintained at 37 °C with 5% CO_2_.

### 4.4. MTT Assay

The effect of the 14 compounds isolated from *N. jatamansi* on cell viability was measured using the MTT assay. To measure cell viability, cells were maintained at a density of 2 × 10^4^ cells in 48-well plates. HaCaT keratinocytes were cultured for 24 h and treated with each compound at concentrations of 20 and 40 μM. All compounds were dissolved in DMSO, and the treatment concentration of DMSO was maintained at 0.1% or less, relative to the medium. Thereafter, they were treated with MTT (0.5 mg/mL) for 1 h to form formazan. Formazan was then dissolved in DMSO, and the absorbance was measured at 540 nm using an ELISA microplate reader (Molecular Devices, San Jose, CA, USA).

### 4.5. IL-6, IL-8, and RANTES Detection in the Cell Supernatant

HaCaT keratinocytes were cultured at a density of 2 × 10^5^ cells/well in a 24-well plate. Thereafter, 14 compounds (10–40 μM) isolated from *N. jatamansi* were pre-treated for 3 h and stimulated with TNF-α/IFN-γ (each at 10 ng/mL) for 24 h. Subsequently, following the manufacturer’s guidelines, the levels of IL-6, IL-8, and RANTES in the cell culture supernatant were measured using an ELISA kit. Dexamethasone (40 μM), which has been reported to have an anti-inflammatory effect on HaCaT keratinocytes [37], was used as the positive control group to investigate the effect of IL-6 inhibition.

### 4.6. Western Blot Analysis

Cycloolivil was used to pre-treat HaCaT keratinocytes at concentrations of 10–40 μM for 24 h. The cells were stimulated with TNF-α/IFN-γ (each at 10 ng/mL) to induce an inflammatory response. Subsequently, western blot analysis was performed, and the proteins were separated using sodium dodecyl sulfate-polyacrylamide gel electrophoresis. Subsequently, the proteins were transferred onto nitrocellulose membranes. Membranes were blocked with 5% skim milk for 1 h and incubated overnight at 4 °C with primary antibodies against COX-2 (diluted 1:1000). Thereafter, the cells were incubated for 1 h at room temperature (25 °C) using a secondary antibody conjugated to horseradish peroxidase diluted at 1:5000. Finally, the membrane was washed with tris-buffered saline and Polysorbate 20 solution, and specific proteins were identified using an enhanced chemiluminescence solution. The optical density of the protein bands was analyzed using ImageJ software version 6.0 (National Institutes of Health, Rockville, MD, USA).

### 4.7. Extraction of Total, Nuclear, and Cytosolic Proteins

To analyze the levels of p-IκBα, IκBα, p65, p-JAK2, p-STAT1, and p-STAT3, HaCaT keratinocytes were pre-treated with cycloolivil at concentrations of 10–40 μM for 24 h. Subsequently, the cells were stimulated with TNF-α/IFN-γ (each at 10 ng/mL) to induce an inflammatory response, followed by total protein analysis. For nuclear and cytoplasmic protein analyses, the cells were collected and lysed in RIPA buffer. Proteins were extracted using a Nuclear Extraction Kit (Cayman Chemical, Ann Arbor, MI, USA) according to the manufacturer’s instructions.

### 4.8. Statistical Analysis

The results for each group are presented as the mean ± standard deviation (SD) (*n* = 3). One-way analysis of variance was conducted using GraphPad Prism software version 9.0 (GraphPad Software Inc., San Diego, CA, USA). Data were compared using Duncan’s multiple comparison tests. Statistical significance was set at * *p* < 0.05, ** *p* < 0.01, *** *p* < 0.001 compared to the TNF-α/IFN-γ-treated groups.

## 5. Conclusions

In this study, 14 compounds were isolated from *N. jatamansi*, which is traditionally used to treat various diseases, including liver diseases. The isolated compounds were structurally identified as olivil (**1**), cycloolivil (**2**), 2-(3’-hydroxy-5’-ethoxyphenyl)-3-hydroxylmethyl-7-methoxy-2,3-dihydrobenzofuran-5-carboxylic acid (**3**), teuclatriol (**4**), bullatantriol (**5**), 1β,4β,7α-trihydroxyeudesmane (**6**), nardoeudesmol A (**7**), debilon (**8**), nardonoxide (**9**), nardosinone (**10**), 4β-hydroxy-8β-methoxy-10-methylene-2,9-dioxatricyclo[4.3.1.0^3,7^]decane (**11**), jatamanins A (**12**), 4α,5-dimethyl-1,3-dioxo1,2,3,4,4α,5,6,7-octahydronaphthalene (**13**), and kanshone M (**14**) through 1D and 2D NMR. Among the isolated compounds, cycloolivil (**2**) and 2-(3’-hydroxy-5’-ethoxyphenyl)-3-hydroxylmethyl-7-methoxy-2,3-dihydrobenzofuran-5-carboxylic acid (**3**) are the first to be discovered in *N. jatamansi*. Subsequently, we aimed to identify the compound with the strongest inhibitory effect against skin inflammation among the compounds isolated from *N. jatamansi*. Cycloolivil (**2**) and 4β-hydroxy-8β-methoxy-10-methylene-2,9-dioxatricyclo[4.3.1.0^3,7^]decane (**11**) exhibited the strongest anti-inflammatory effect on IL-6 production in HaCaT keratinocytes induced by TNF-α/IFN-γ. Both compounds effectively suppressed production of the inflammatory cytokines IL-8 and RANTES, and cycloolivil (**2**) exhibited excellent anti-inflammatory effects. In conclusion, our findings confirmed that cycloolivil (**2**) derived from *N. jatamansi* exhibits an inhibitory effect against skin inflammation by inhibiting the targeting the JAK/STAT and NF-κB signaling pathways. Our study identifies cycloolivil (**2**), for the first time, as a highly effective compound against skin inflammation, suggesting its potential as a therapeutic agent for atopic dermatitis.

## Figures and Tables

**Table 1:** Half-maximal inhibitory concentration (IC_50_) values of *N. jatamansi* compounds with significant inhibitory effects against TNF-α/IFN-γ-induced IL-6 production in HaCaT keratinocytes.

Compound	IC_50_ (IL-6)
Cycloolivil (**2**)	31.05 ± 0.93 μM
4β-hydroxy-8β-methoxy-10-methylene-2,9-dioxatricyclo[4.3.1.0^3,7^]decane (**11**)	28.25 ± 0.21 μM

## Data Availability

The data presented in this study are available on request from the corresponding author.

## Supplementary Materials

The following supporting information can be downloaded at: https://www.mdpi.com/article/10.3390/ijms25063342/s1.
