# Supplementary material for: Cycloolivil Isolated from Nardostachys jatamansi Inhibits TNF-α/IFN-γ-Induced Chemokine Production by Blocking NF-κB and JAK/STAT Activation in HaCaT Keratinocytes
Source: Int J Mol Sci. 2024 Mar 15;25(6):3342. doi: 10.3390/ijms25063342 (PMC10969846; doi:10.3390/ijms25063342)
Supplement: Supplementary file 1 [file ijms-25-03342-s001.zip › ijms-2879527-supplementary.pdf]

Supplementary information

# Cycloolivil Isolated from *Nardostachys jatamansi* Inhibits TNF-α/IFN-γ-Induced Chemokine Production by Blocking NF-κB and JAK/STAT Activation in HaCaT Keratinocytes

**Chi-Su Yoon [1,†], Hwan Lee [2,†], Zhiming Liu [2], Linsha Dong [2], Gyoyoung Lee [2], Nayeon Kim [2], Hyuncheol Oh [1,*] and Dong-Sung Lee [2,*]**

[1] College of Pharmacy, Wonkwang University, Iksan 54538, Republic of Korea
[2] Research Institute of Pharmaceutical Sciences, College of Pharmacy, Chosun University, Gwangju 61452, Republic of Korea
* Correspondence: hoh@wonkwang.ac.kr (H.O.); dslee2771@chosun.ac.kr (D.-S.L.)
† These authors contributed equally to this work.

# Contents

**Materials and Methods.**

**Figure S1.** $^{1}H$ NMR spectrum of compound **1**.

**Figure S2.** $^{13}C$ NMR spectrum of compound **1**.

**Figure S3.** HRESI-MS spectrum of compound **1**.

**Figure S4.** $^{1}H$ NMR spectrum of compound **2**.

**Figure S5.** $^{13}C$ NMR spectrum of compound **2**.

**Figure S6.** HRESI-MS spectrum of compound **2**.

**Figure S7.** HMQC NMR spectrum of compound **2**.

**Figure S8.** COSY NMR spectrum of compound **2**.

**Figure S9.** HMBC spectrum of compound **2**.

**Figure S10.** $^{1}H$ NMR spectrum of compound **3**.

**Figure S11.** $^{13}C$ NMR spectrum of compound **3**.

**Figure S12.** HRESI-MS spectrum of compound **3**.

**Figure S13.** $^{1}H$ NMR spectrum of compound **7**.

**Figure S14.** $^{13}C$ NMR spectrum of compound **7**.

**Figure S15.** HRESI-MS spectrum of compound **7**.

**Figure S16.** $^{1}H$ NMR spectrum of compound **9**.

**Figure S17.** $^{13}C$ NMR spectrum of compound **9**.

**Figure S18.** HRESI-MS spectrum of compound **9**.

**Figure S19.** $^{1}H$ NMR spectrum of compound **10**.

**Figure S20.** $^{13}C$ NMR spectrum of compound **10**.

**Figure S21.** HRESI-MS spectrum of compound **10**.

**Figure S22.** $^{1}H$ NMR spectrum of compound **11**.

**Figure S23.** $^{13}C$ NMR spectrum of compound **11**.

**Figure S24.** HRESI-MS spectrum of compound **11**.

**Figure S25.** HMQC NMR spectrum of compound **11**.

**Figure S26.** COSY NMR spectrum of compound **11**.

**Figure S27.** HMBC spectrum of compound **11**.

**Figure S28.** $^{1}H$ NMR spectrum of compound **13**.

**Figure S29.** $^{13}C$ NMR spectrum of compound **13**.

**Figure S30.** HRESI-MS spectrum of compound **13**.

**Figure S31.** HPLC chromatogram of compound **2**.

**Figure S32.** HPLC chromatogram of compound **11**.

**Materials and Methods**

*HPLC purity checking of bioactive compounds* ***2*** *and* ***11****.*

For HPLC analysis, the YL-9100 PDA system (YoungLin, Korea) was used. The Phenomenex Kinetex C18 HPLC column (4.6 × 150 mm; 5 μm particle size) was used with a flow rate of 0.7 mL/min. The solvent system contained water (0.1% formic acid added) and acetonitrile eluted with a gradient system [10% to 100% AcN (0~20 min); 100% AcN (20~25 min)]. All samples were injected 20 μL in a 20 μL size loop, and **2** and **11** were dissolved in methanol as concentrated 0.2 mg/mL. Compound **2** was detected at 286 nm (Fig.S31), and **11** was detected at 194 nm (Fig.S32).

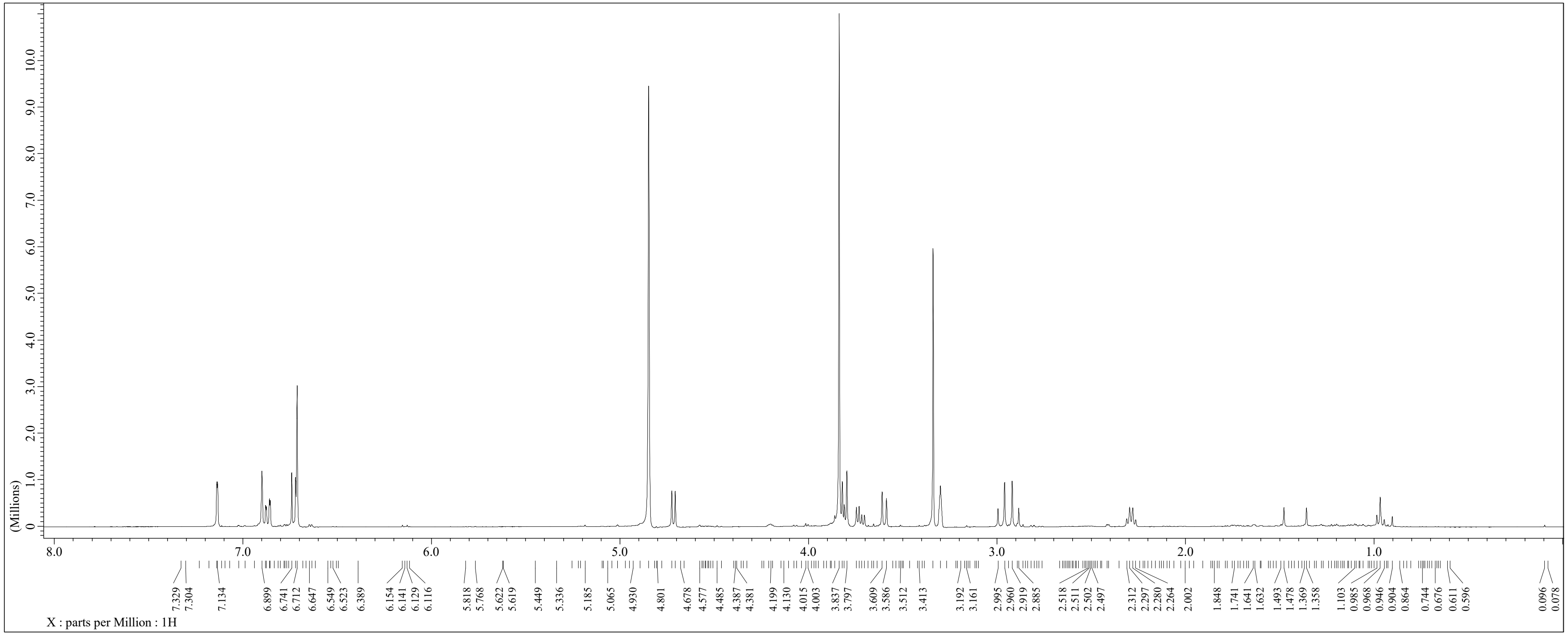


**Figure S1.** $^{1}H$ NMR spectrum of compound **1**.

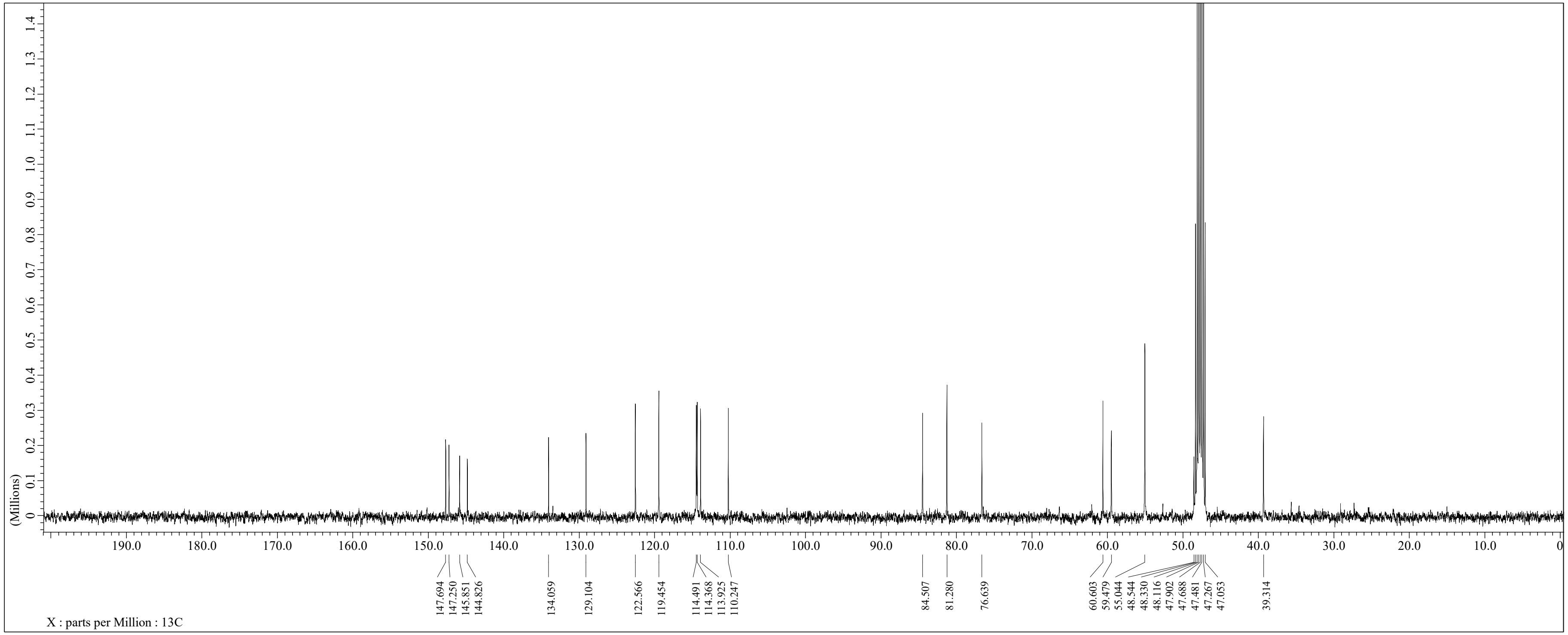


**Figure S2.** $^{13}$C NMR spectrum of compound **1**.

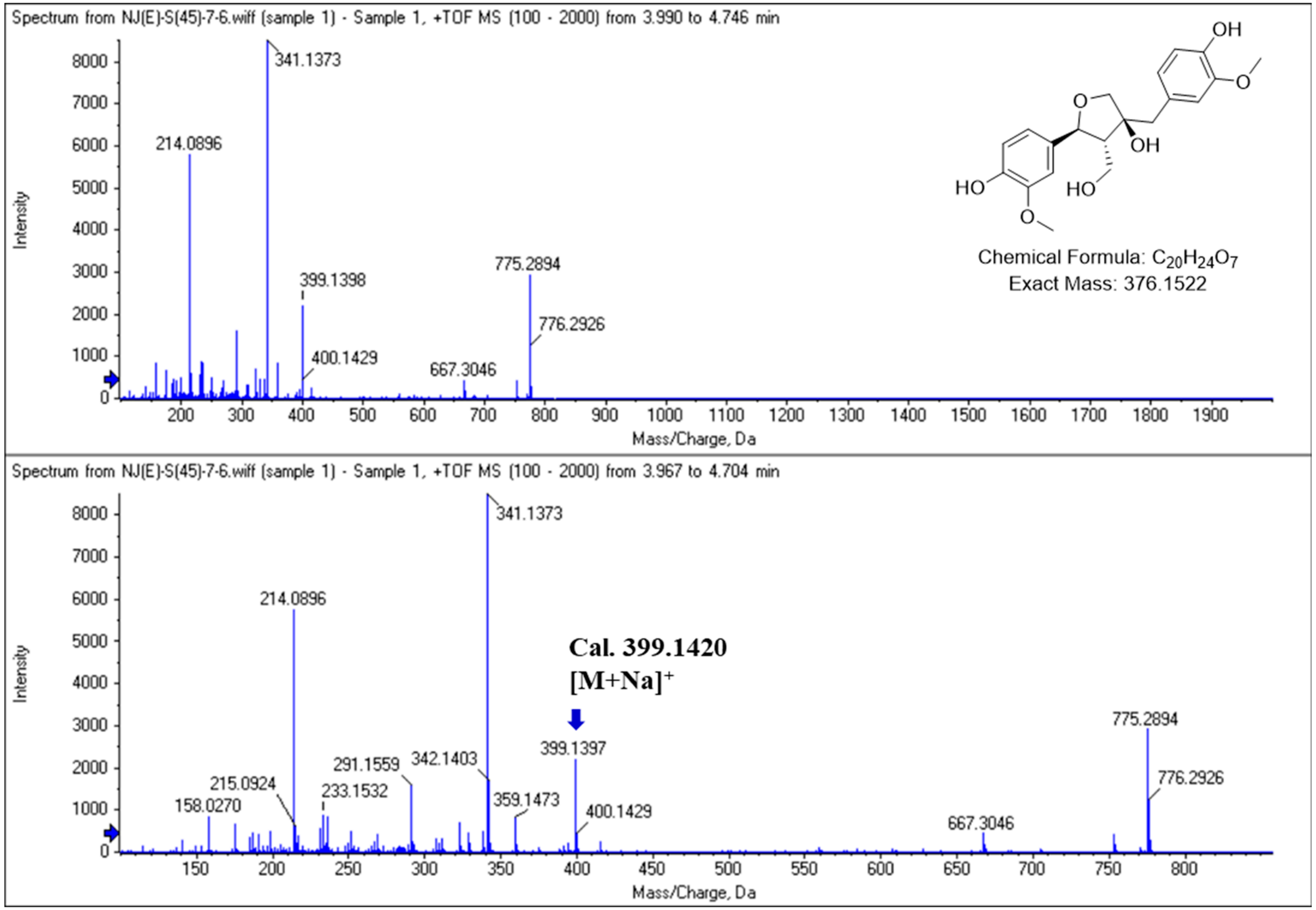


**Figure S3.** HRESI-MS spectrum of compound **1**.

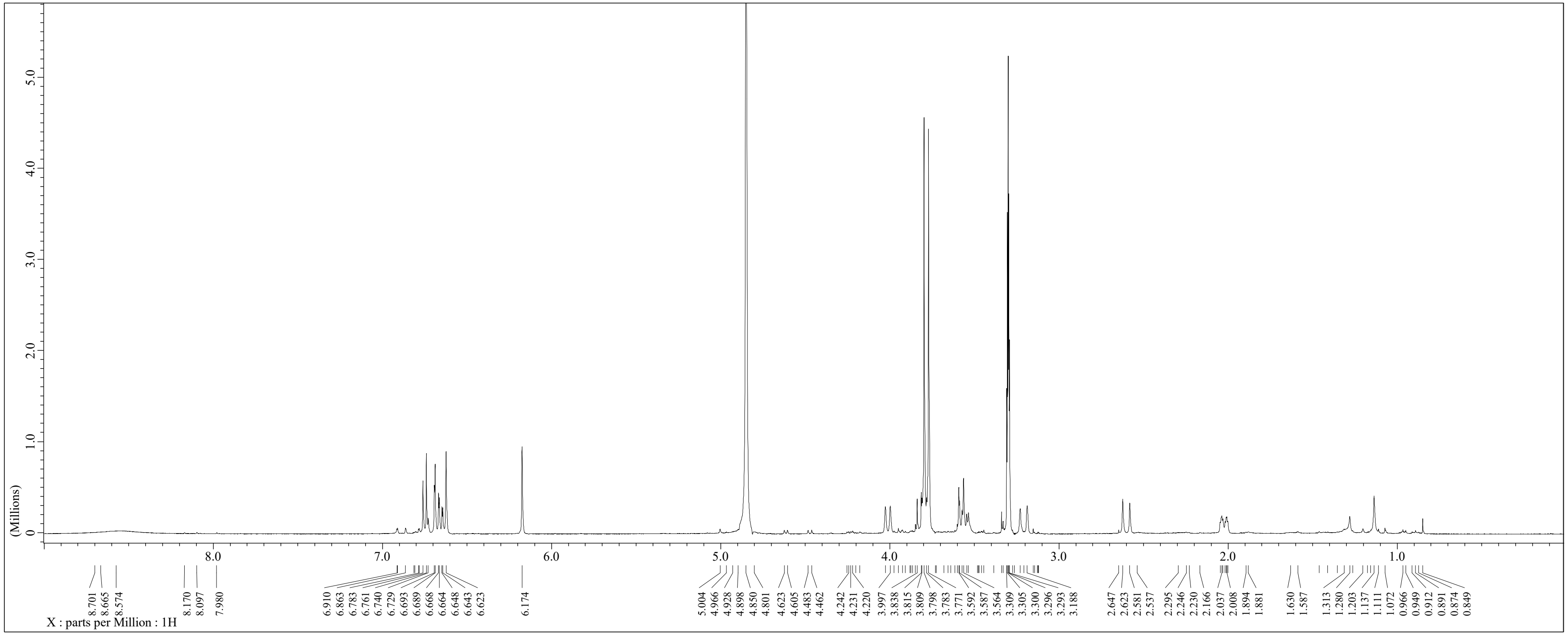


**Figure S4.** $^1$H NMR spectrum of compound **2**.

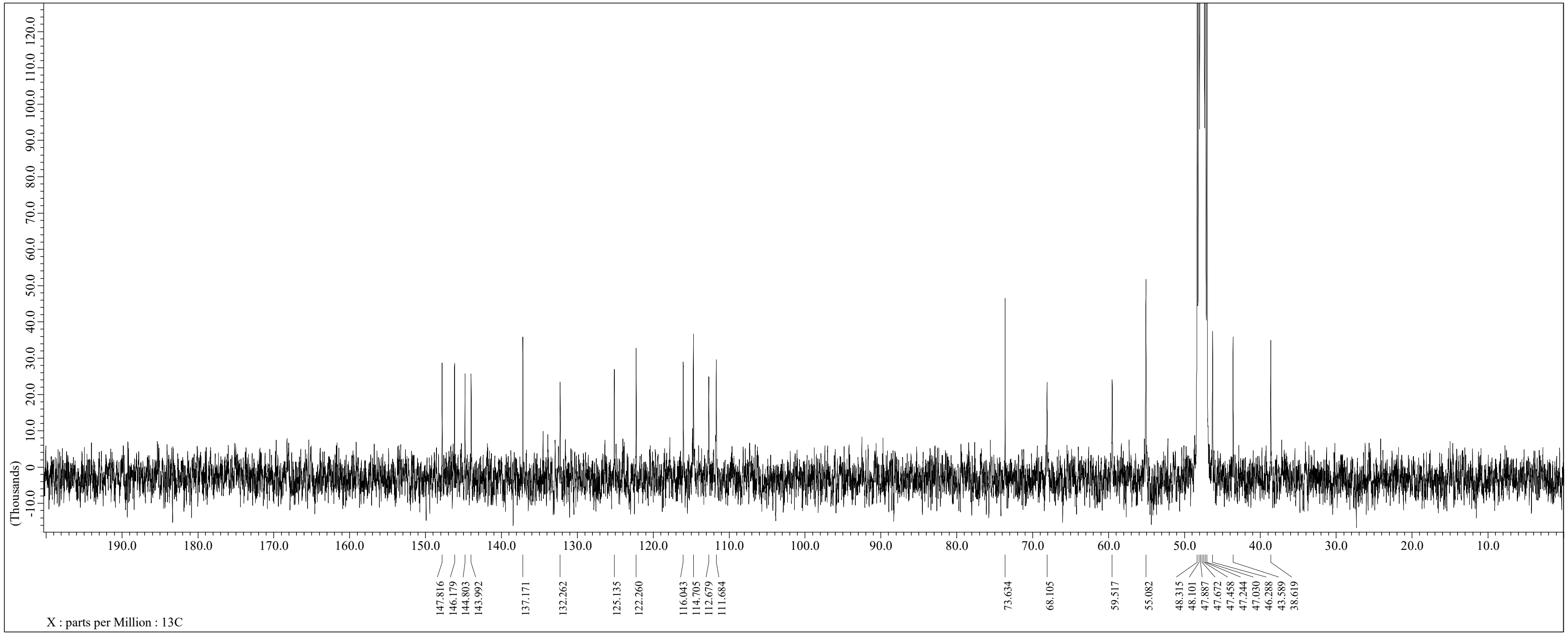


**Figure S5.** $^{13}$C NMR spectrum of compound **2**.

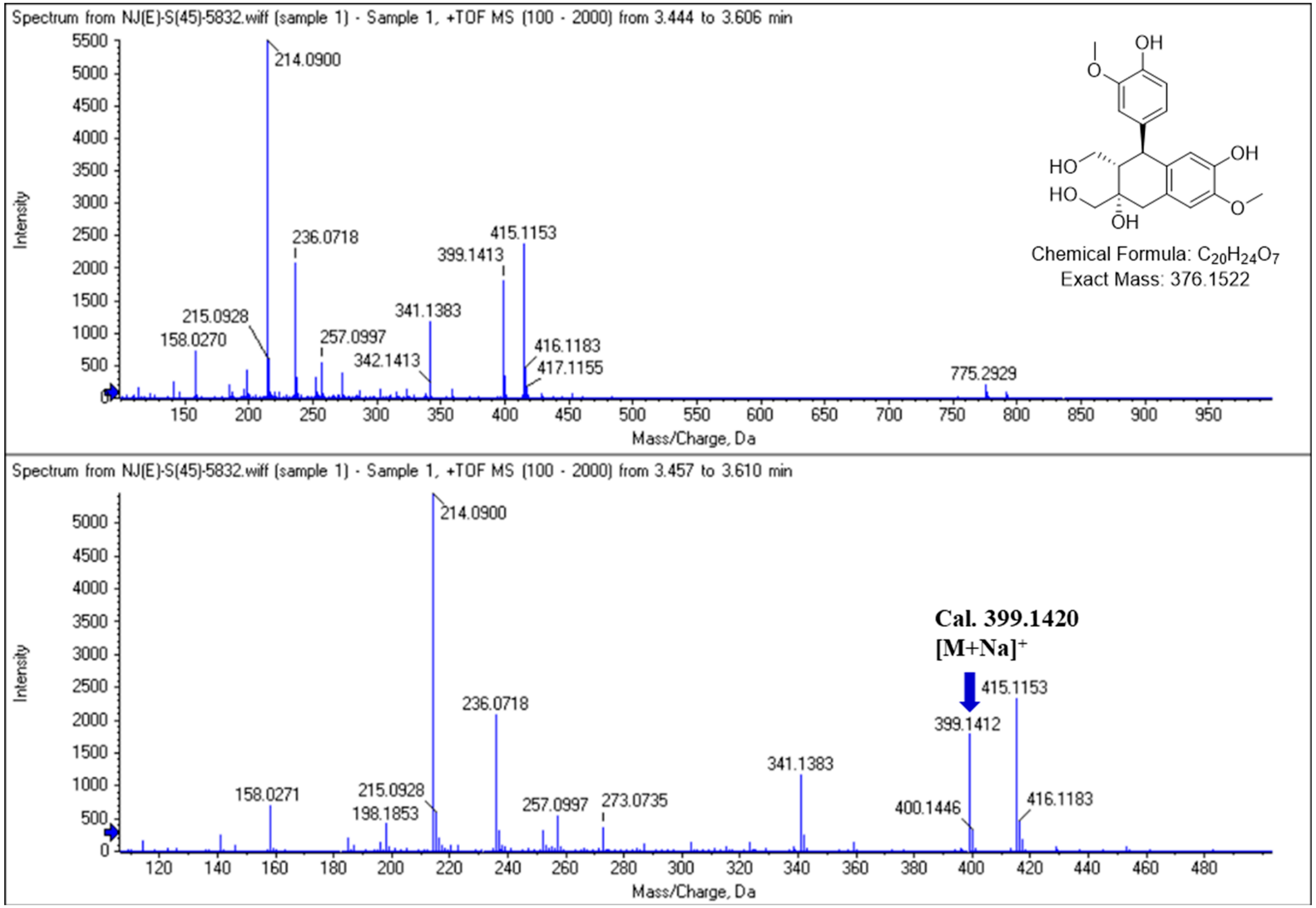


**Figure S6.** HRESI-MS spectrum of compound **2**.

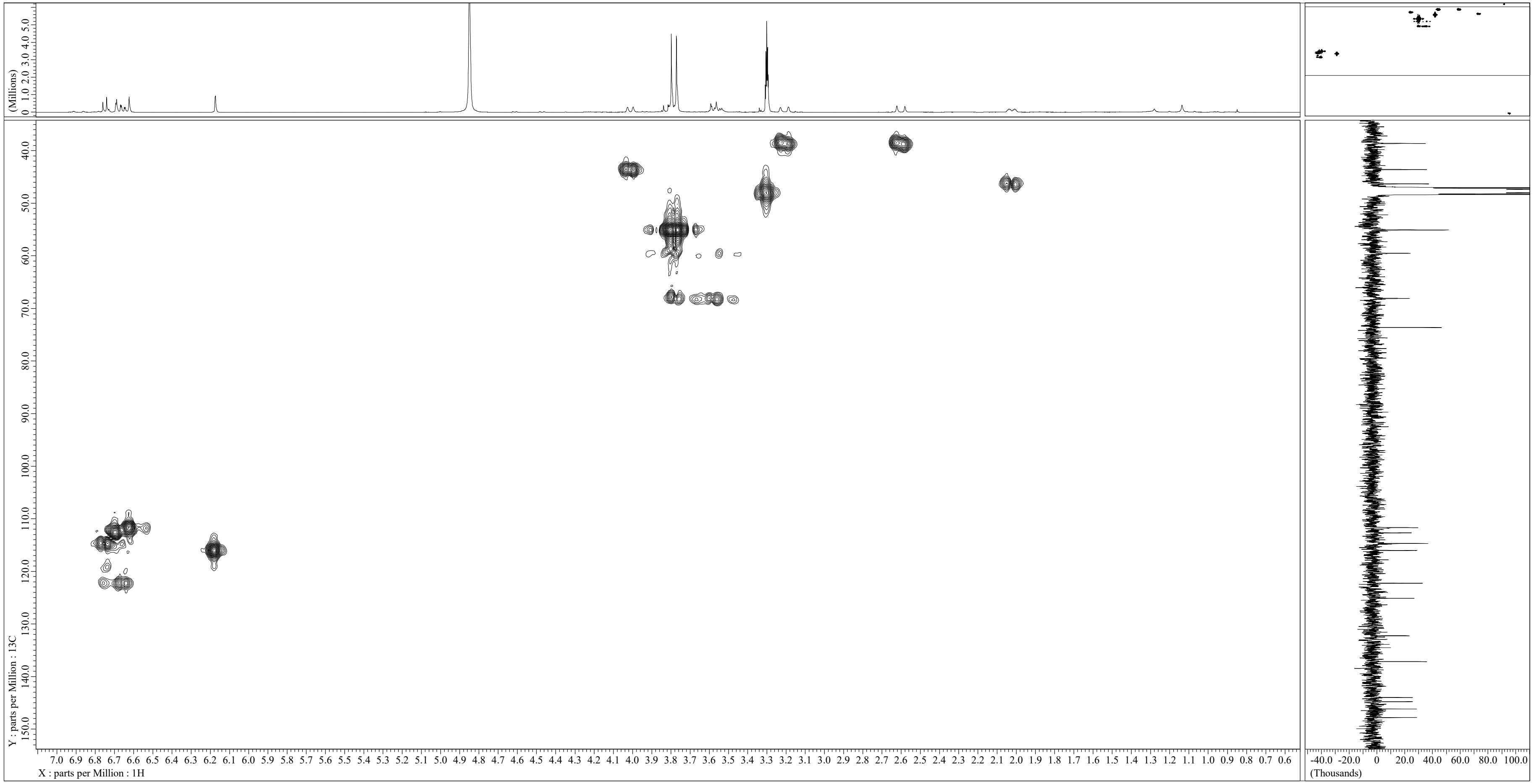


**Figure S7.** HMQC NMR spectrum of compound **2**.

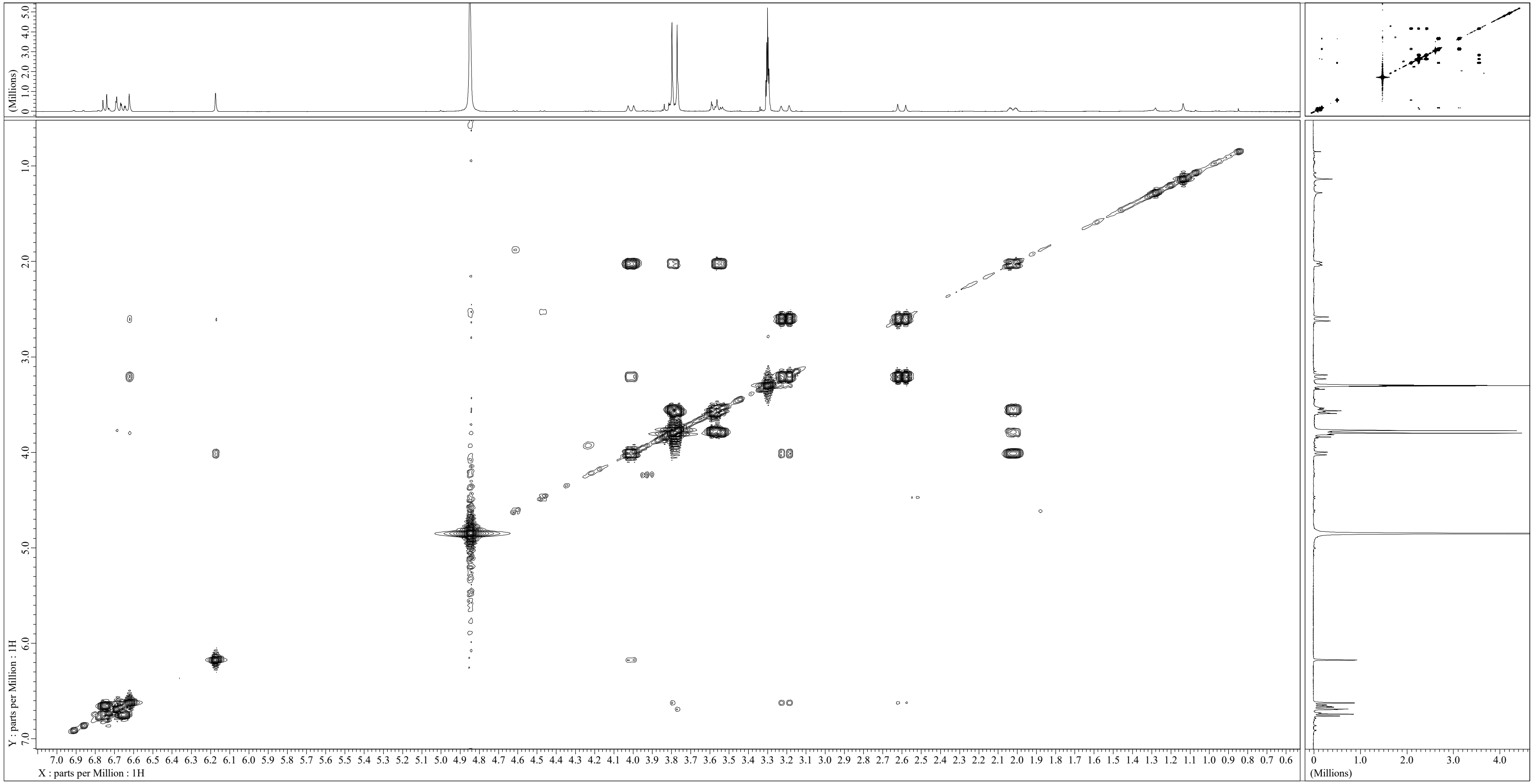


**Figure S8.** COSY NMR spectrum of compound **2**.

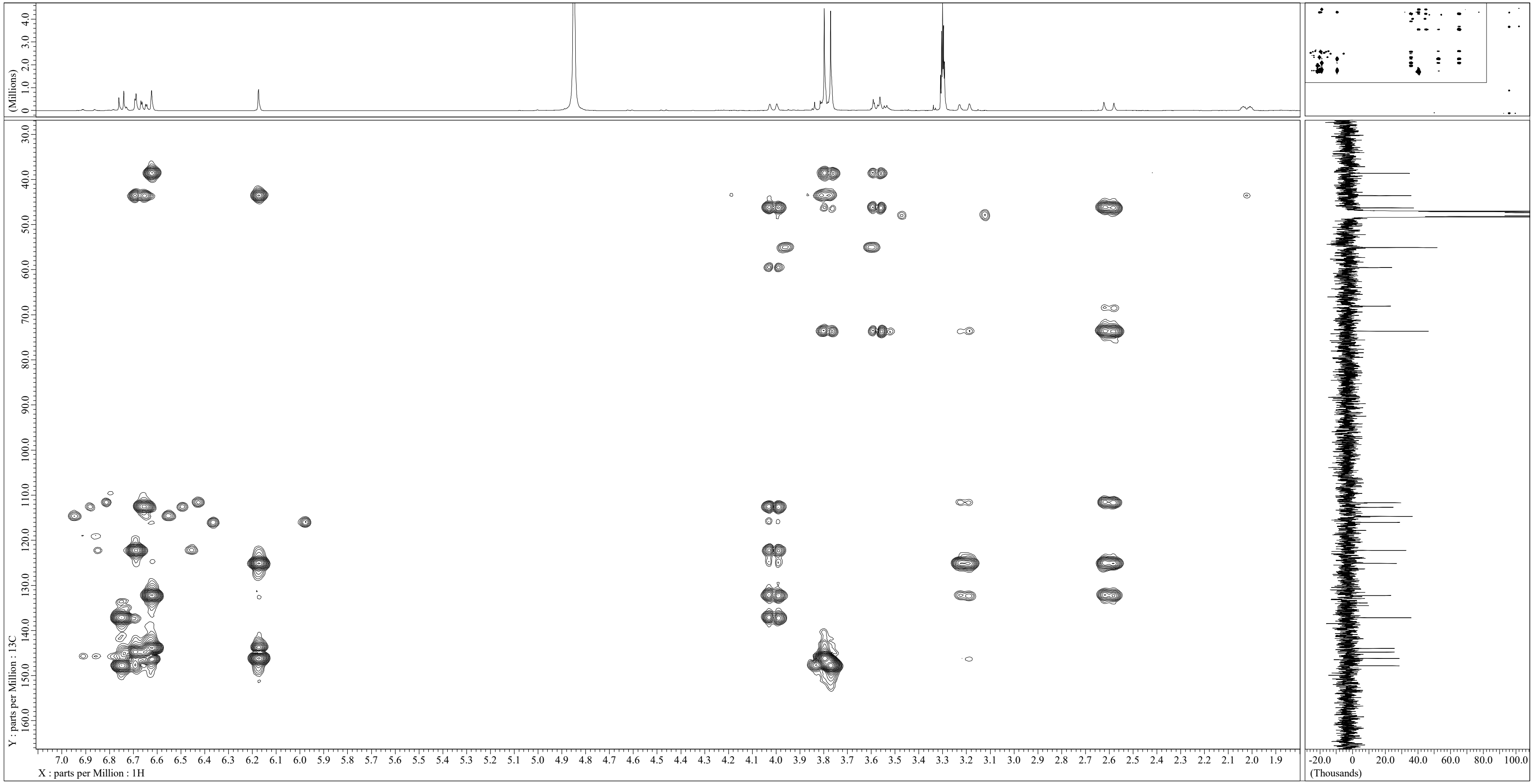


**Figure S9.** HMBC NMR spectrum of compound **2**.

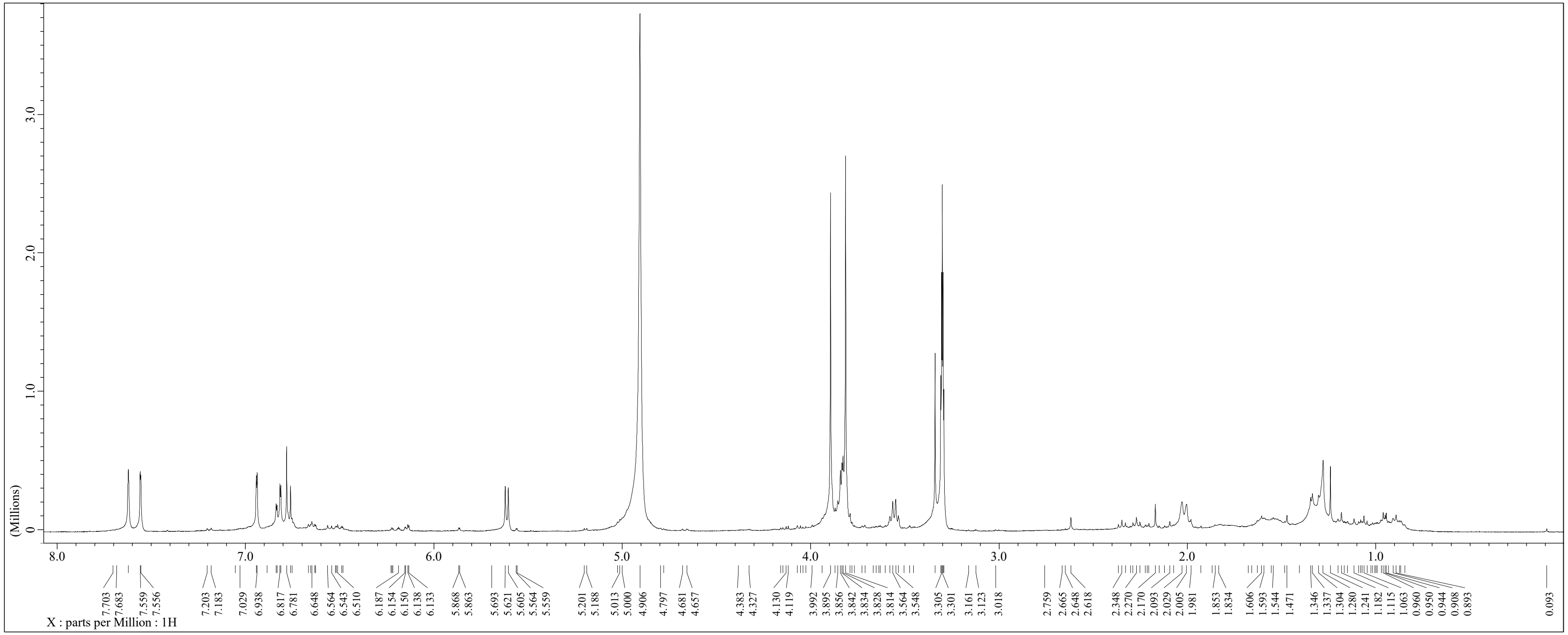


**Figure S10.** $^{1}$H NMR spectrum of compound **3**.

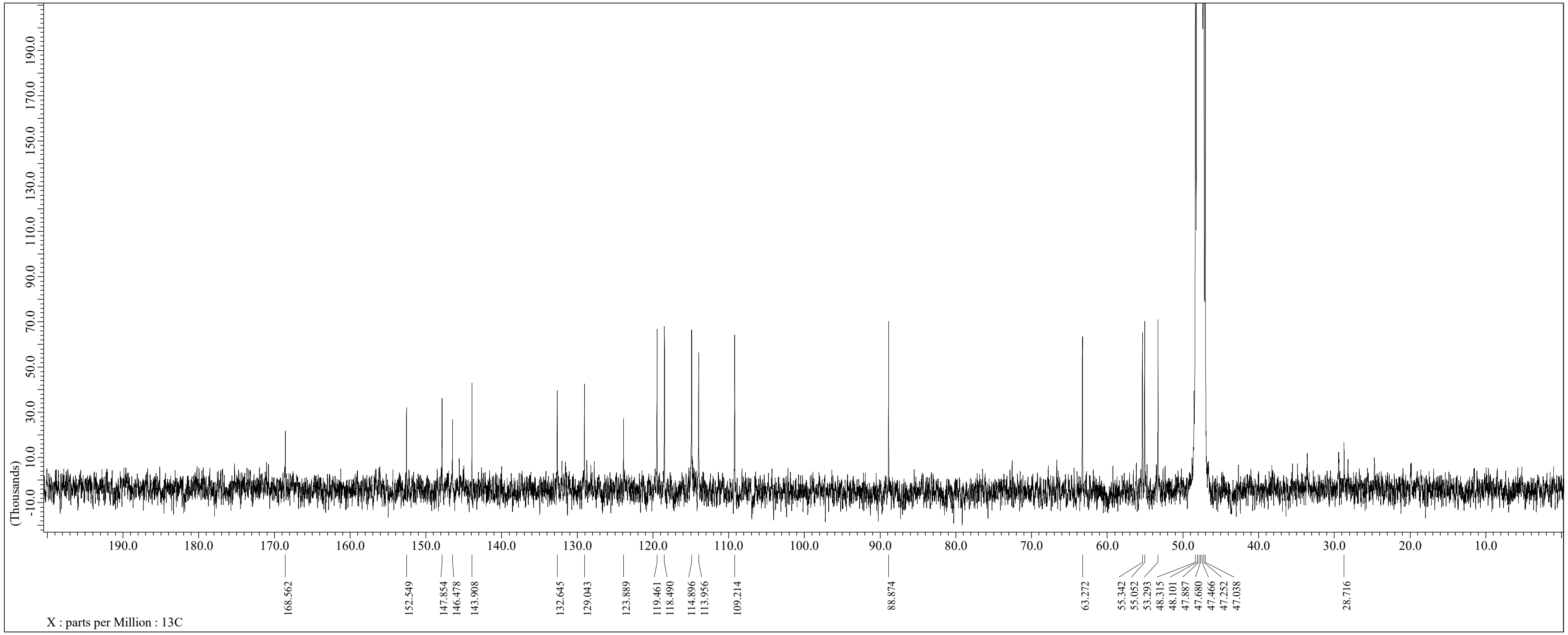


**Figure S11.** $^{13}C$ NMR spectrum of compound **3**.

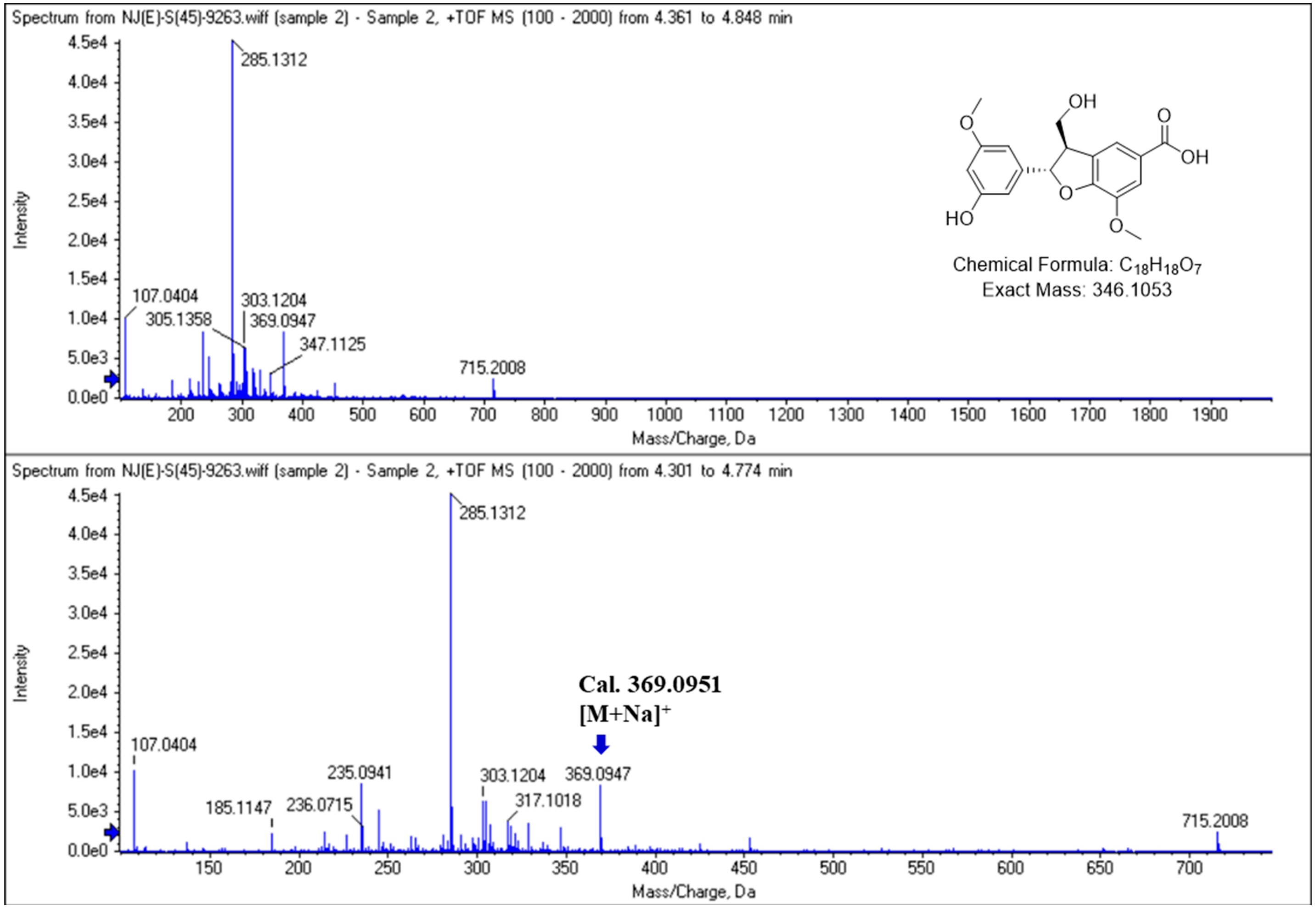


**Figure S12.** HRESI-MS spectrum of compound **3**.

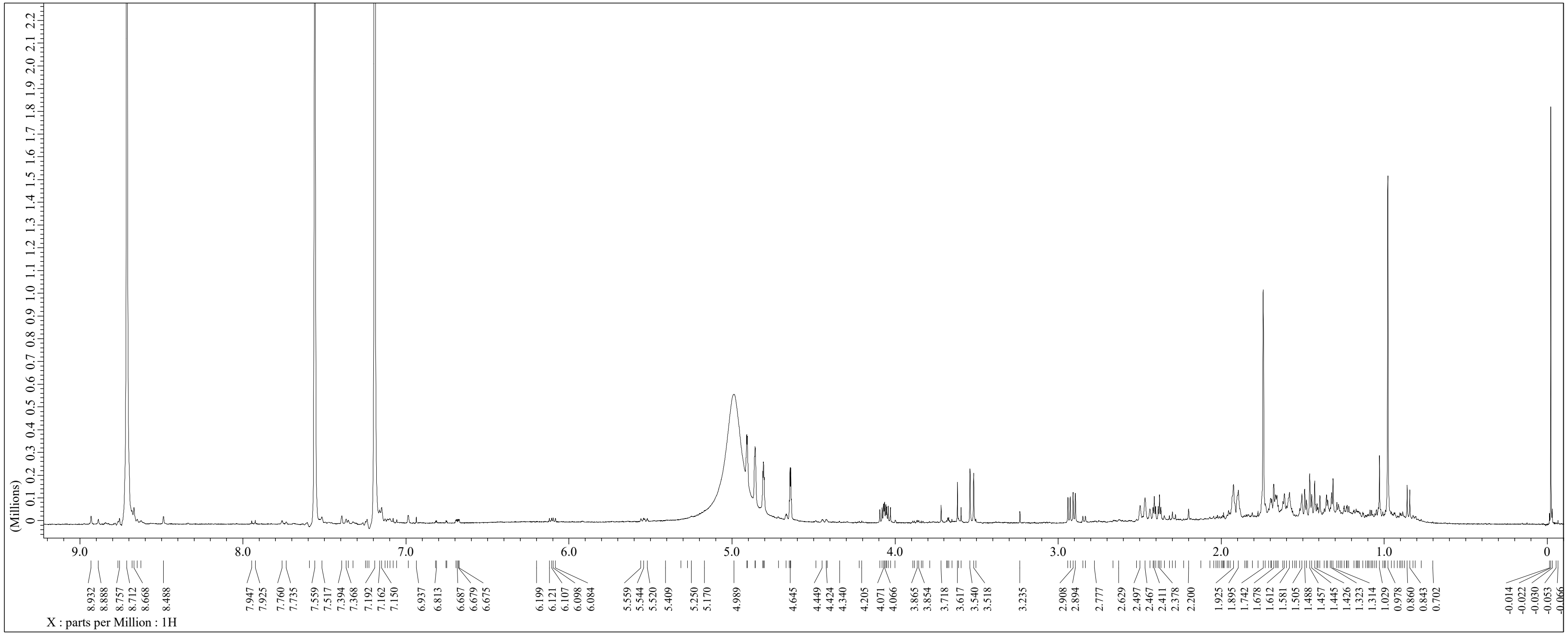

**Figure S13.** $^{1}$H NMR spectrum of compound **7**.

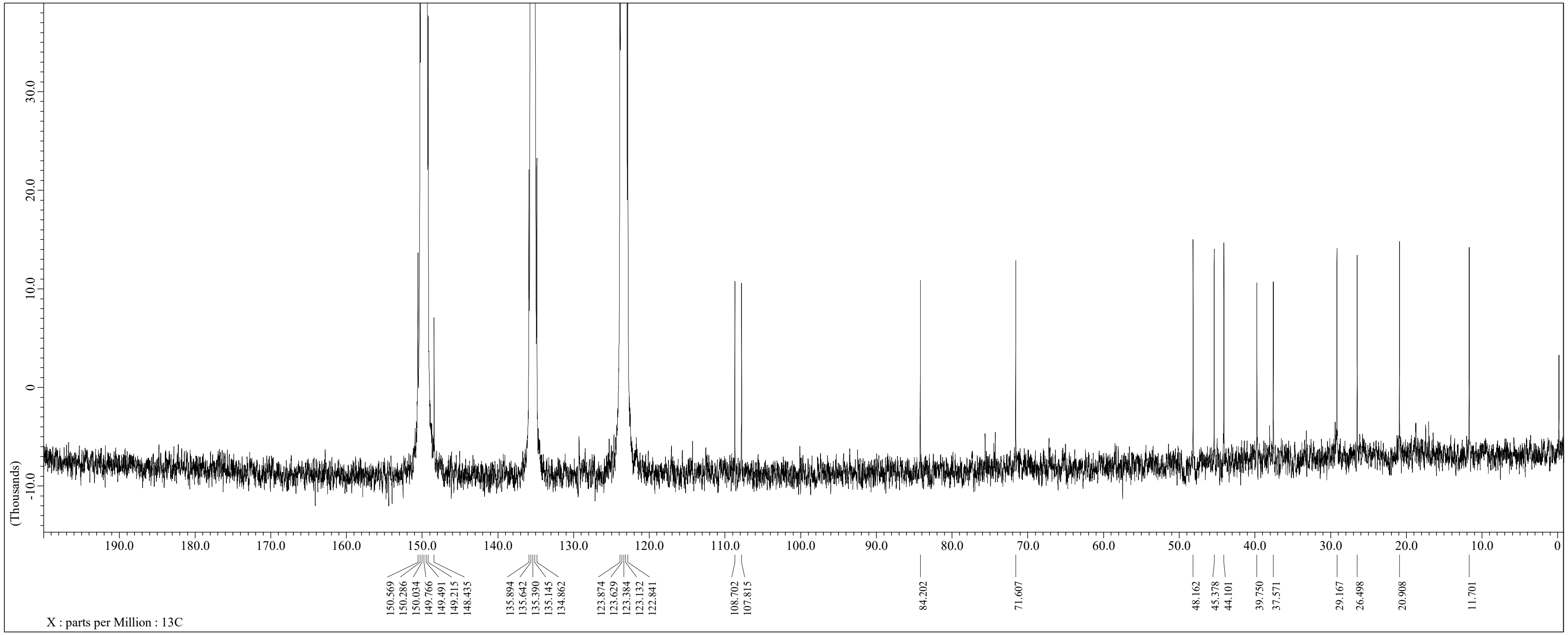


**Figure S14.** $^{13}C$ NMR spectrum of compound 7.

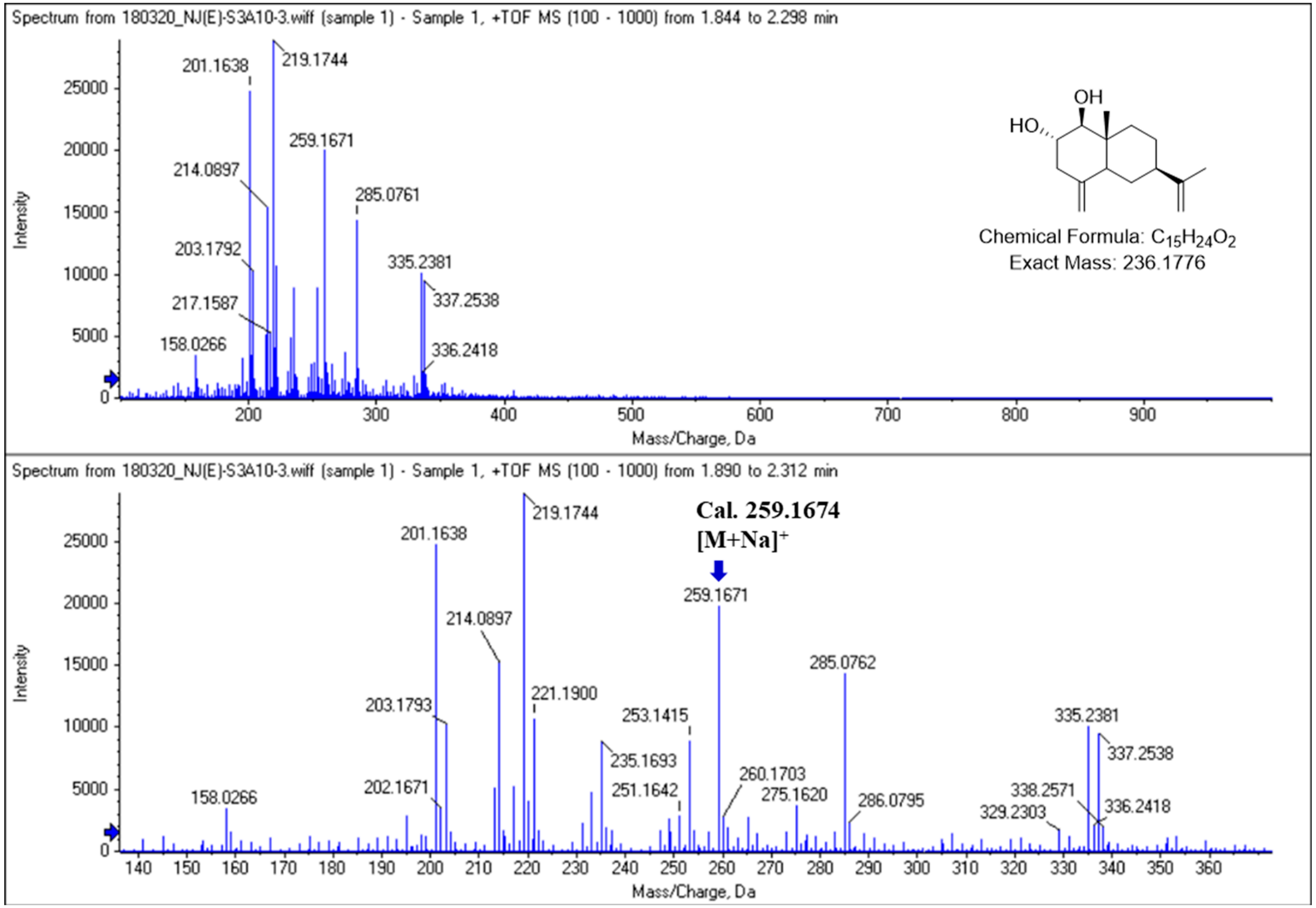


**Figure S15.** HRESI-MS spectrum of compound **7**.

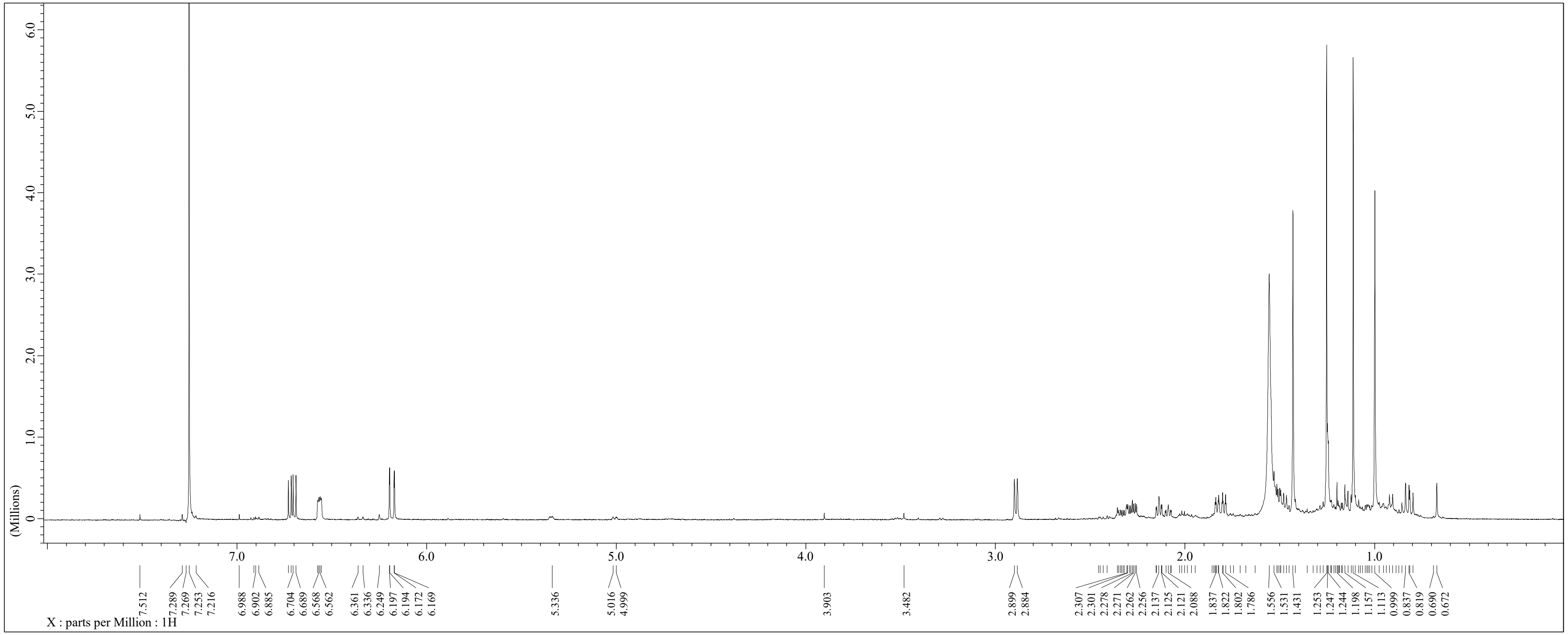

**Figure S16.** $^{1}$H NMR spectrum of compound **9**.

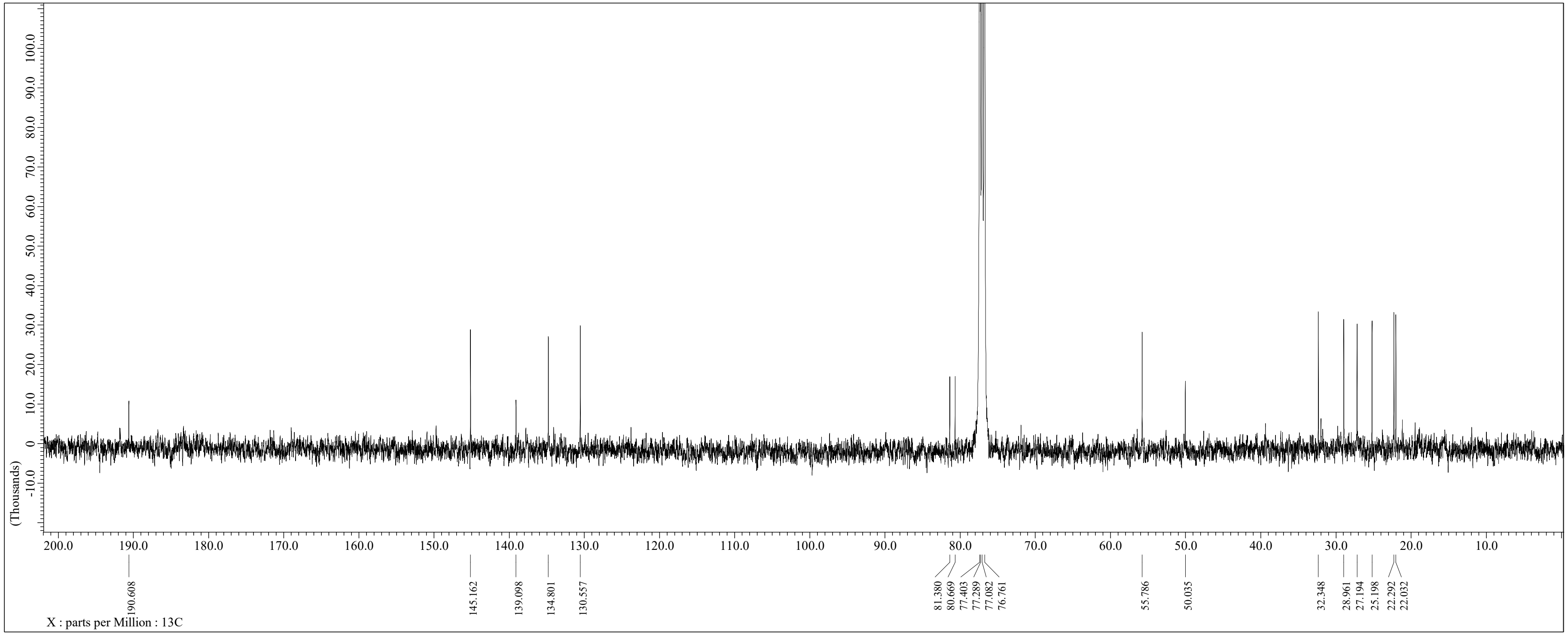


**Figure S17.** $^{13}$C NMR spectrum of compound **9**.

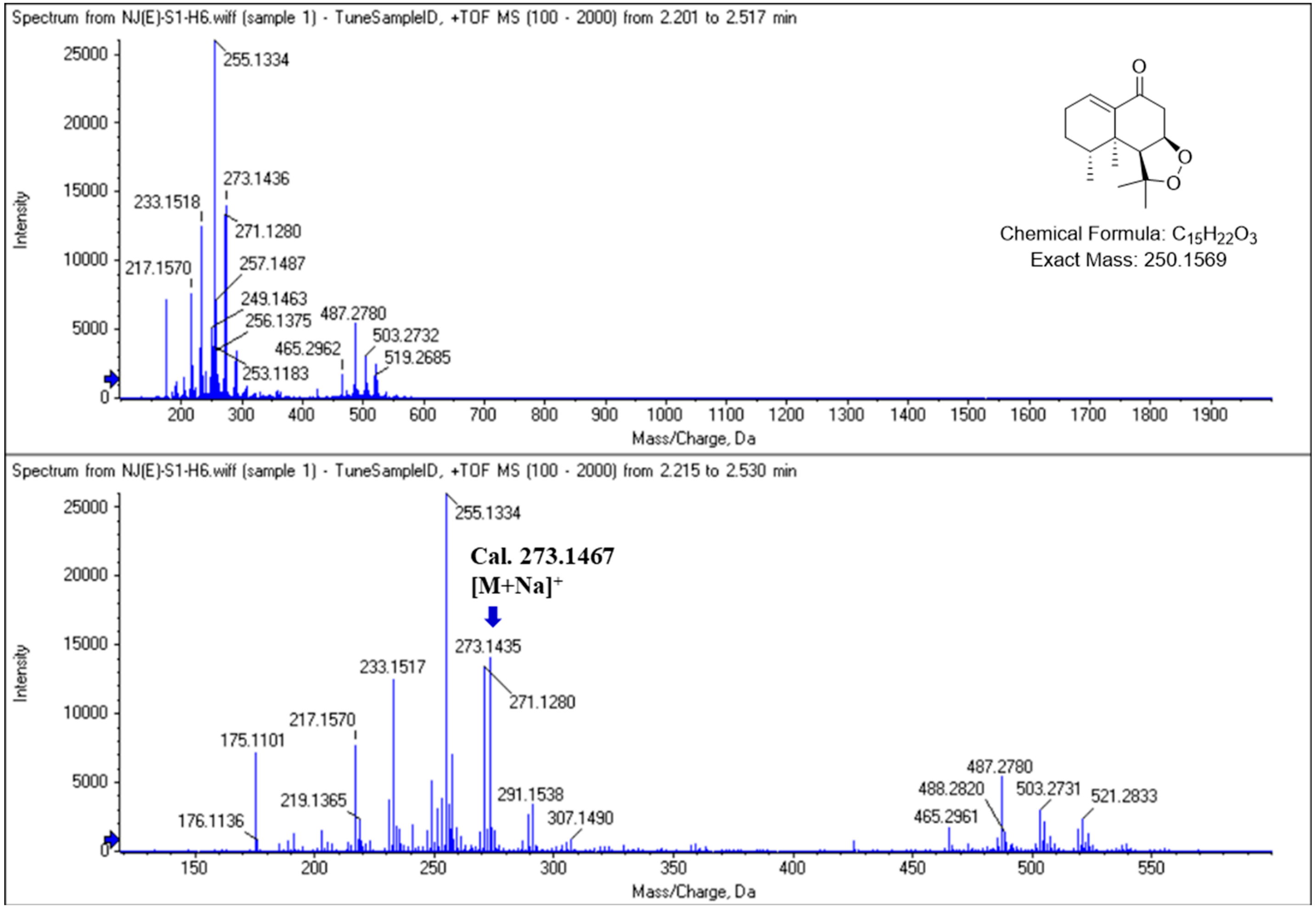


**Figure S18.** HRESI-MS spectrum of compound **9**.

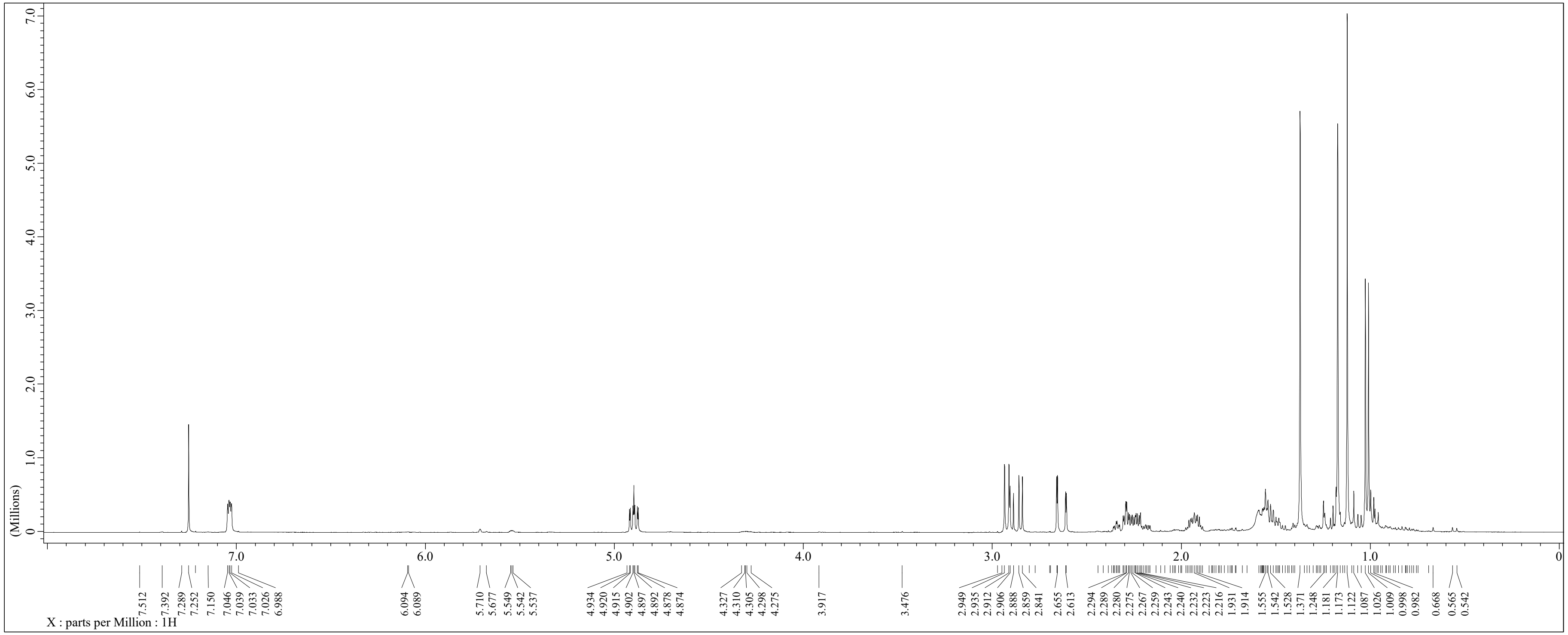

**Figure S19.** $^1$H NMR spectrum of compound **10**.

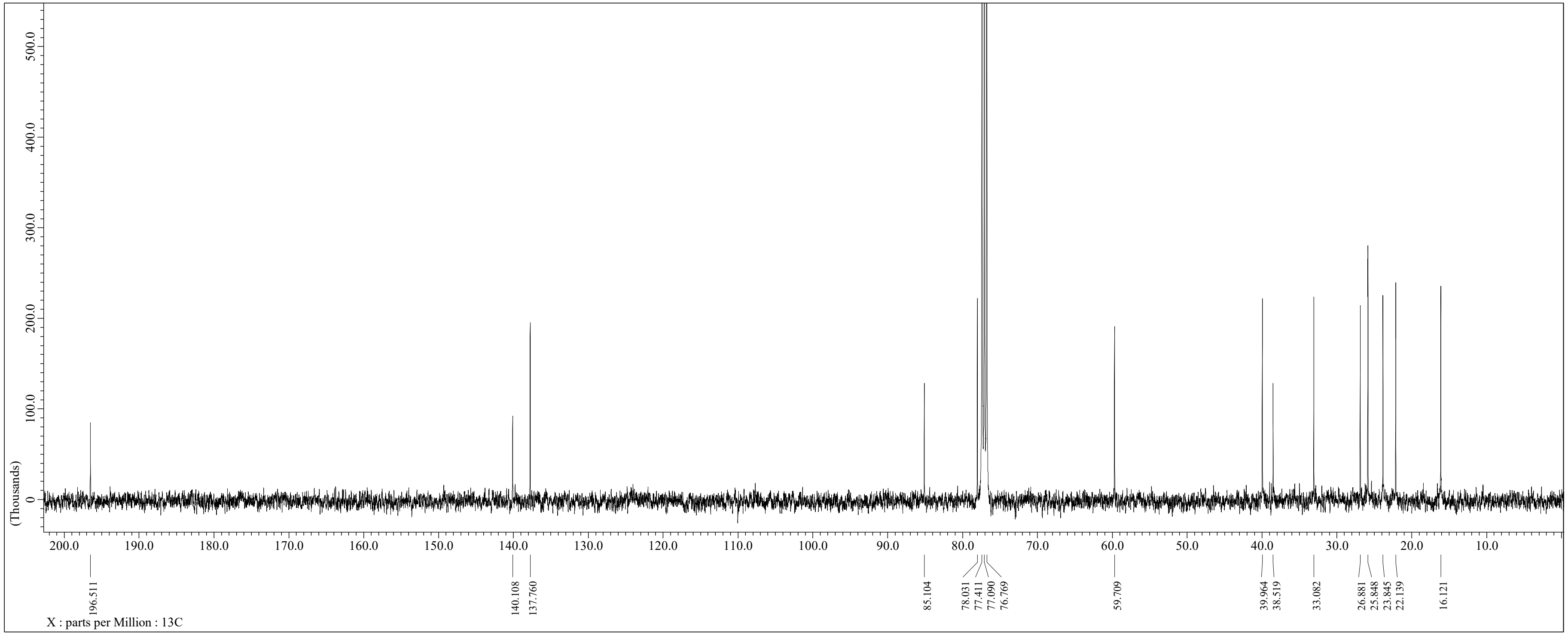


**Figure S20.** $^{13}C$ NMR spectrum of compound **10**.

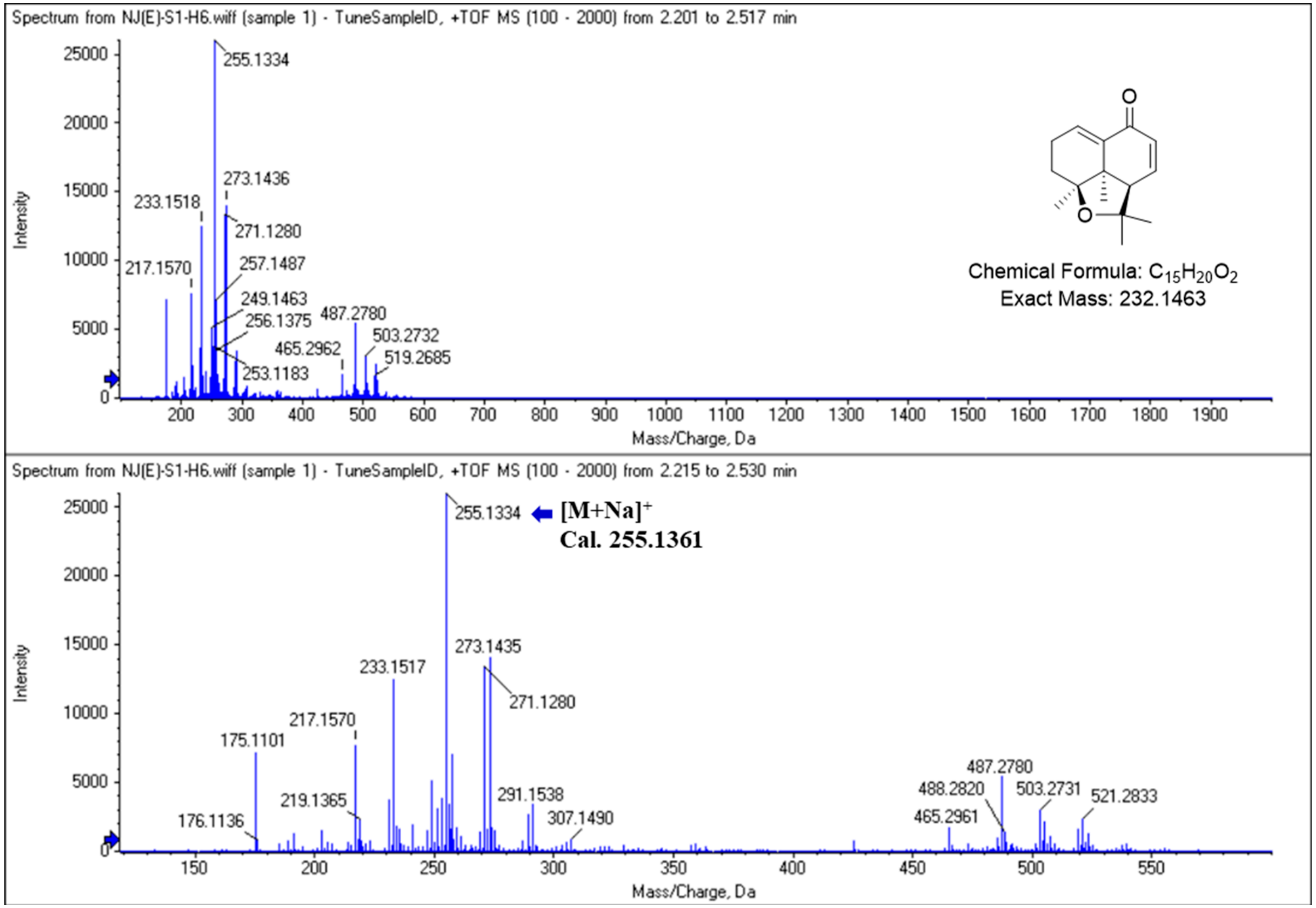


**Figure S21.** HRESI-MS spectrum of compound **10**.

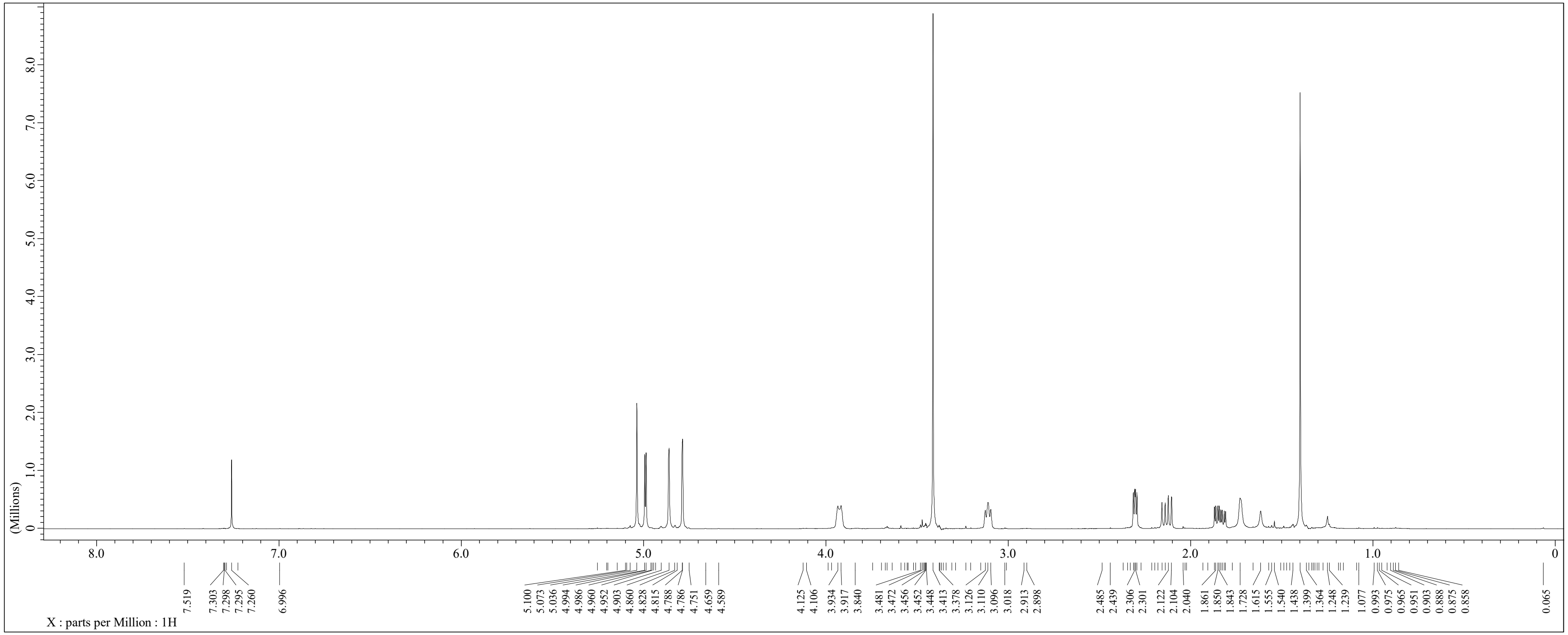

**Figure S22.** $^{1}$H NMR spectrum of compound **11**.

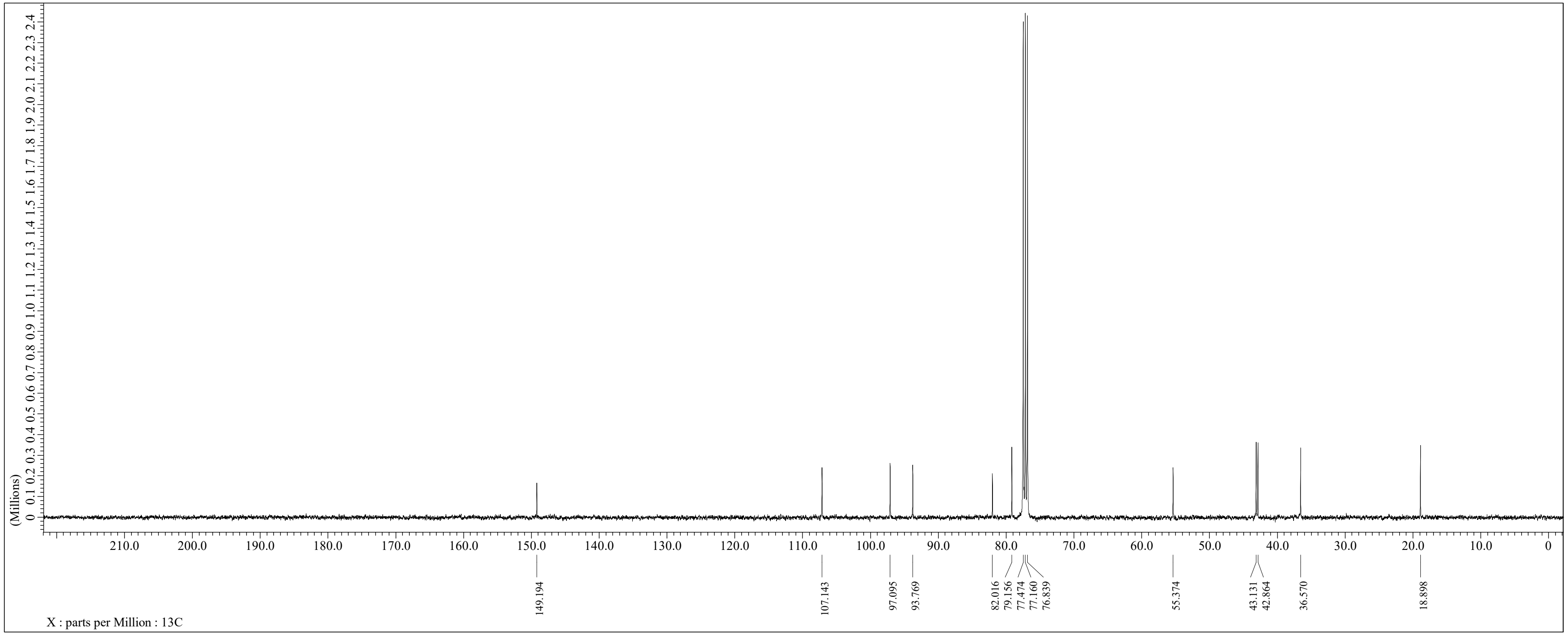


**Figure S23.** $^{13}C$ NMR spectrum of compound **11**.

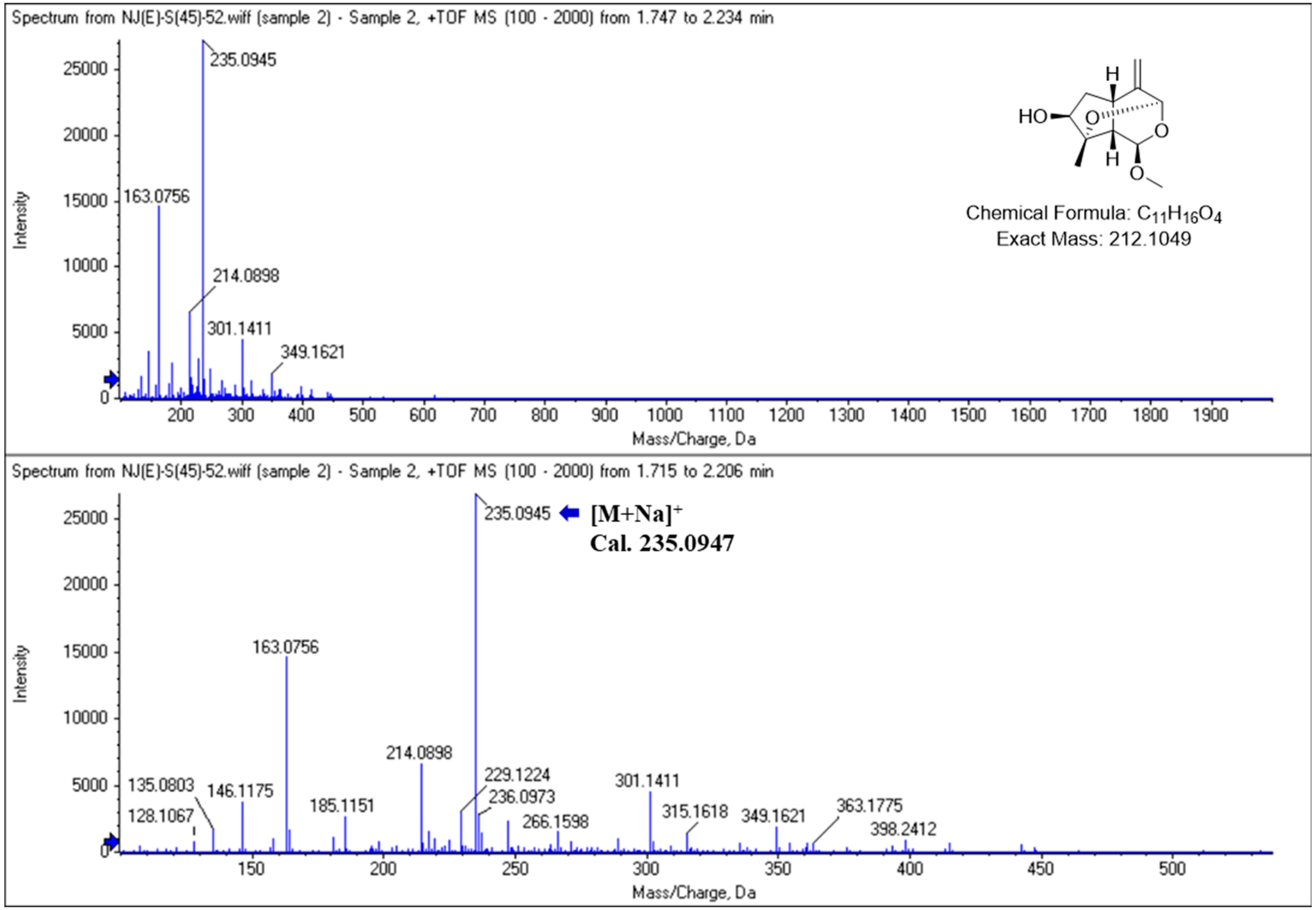


**Figure S24.** HRESI-MS spectrum of compound **11**.

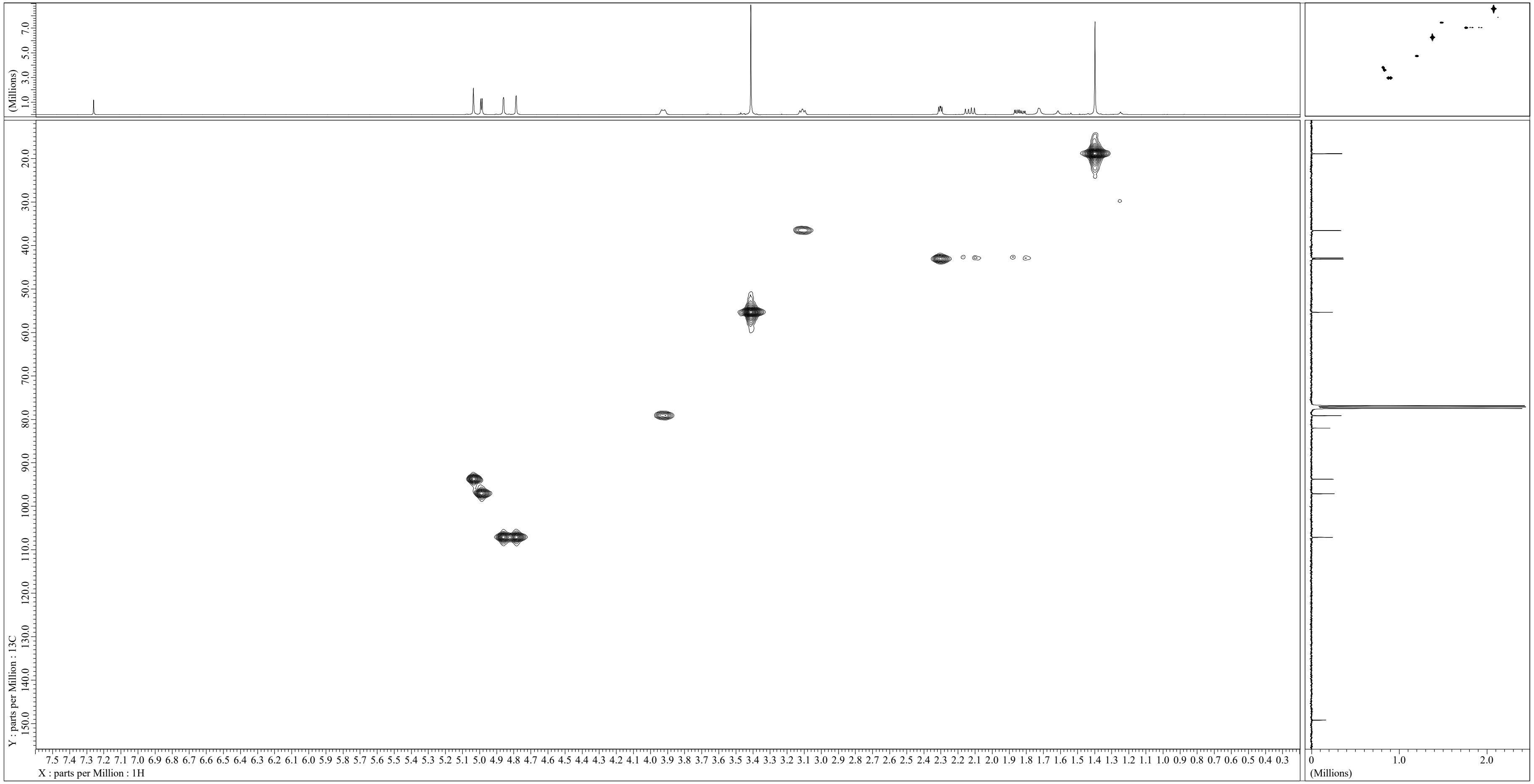


**Figure S25.** HMQC NMR spectrum of compound **11**.

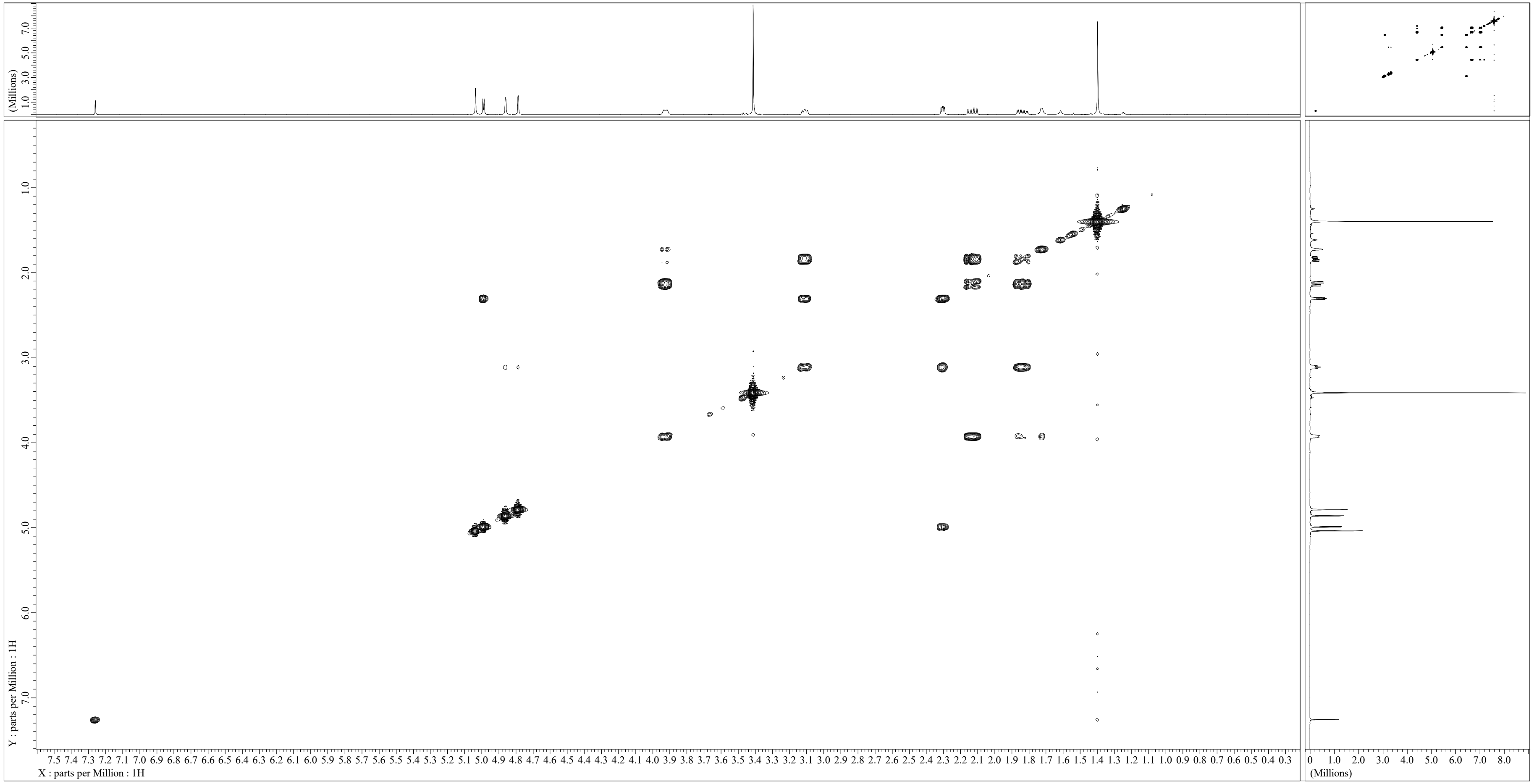


**Figure S26.** COSY NMR spectrum of compound **11**.

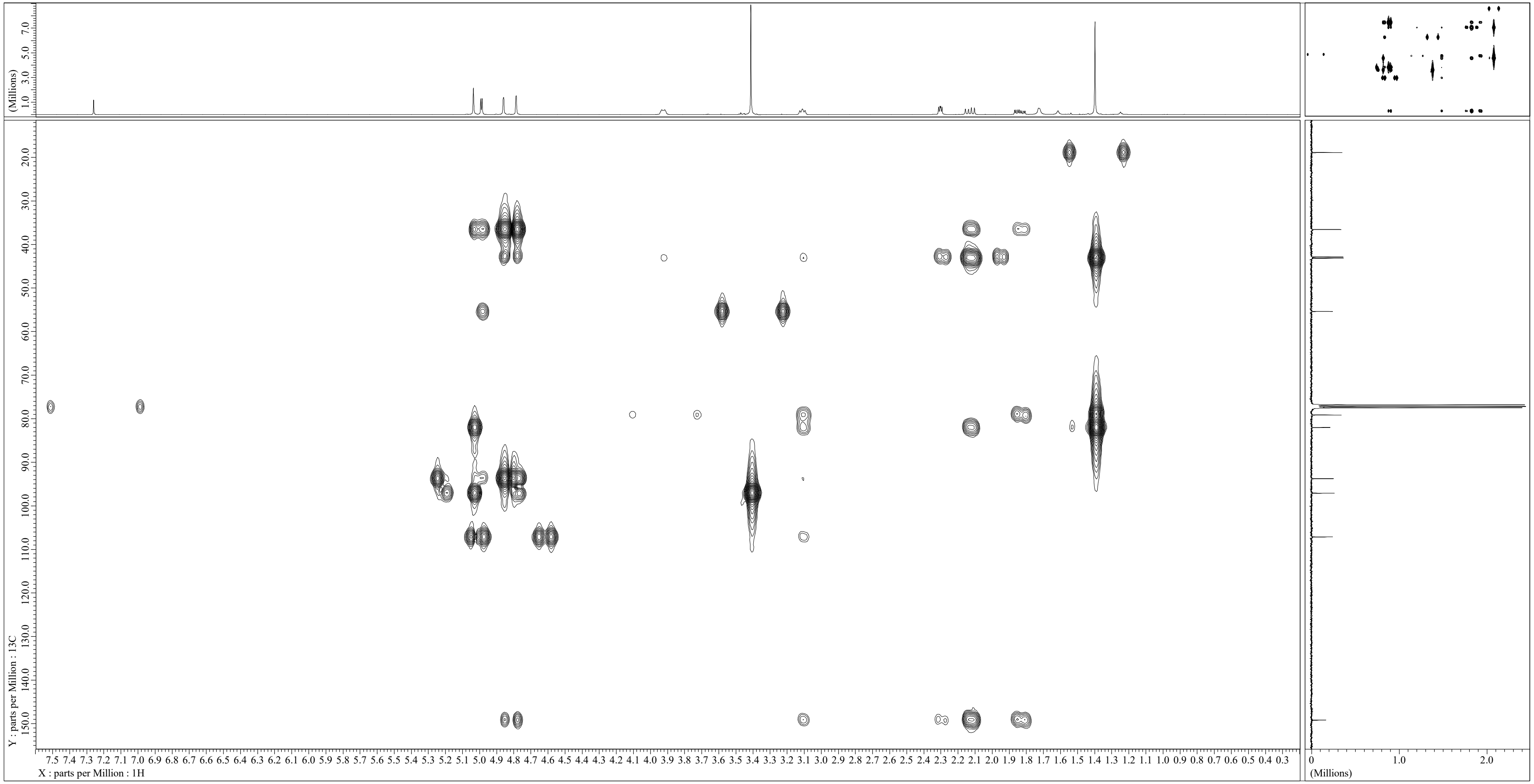


**Figure S27.** HMBC NMR spectrum of compound **11**.

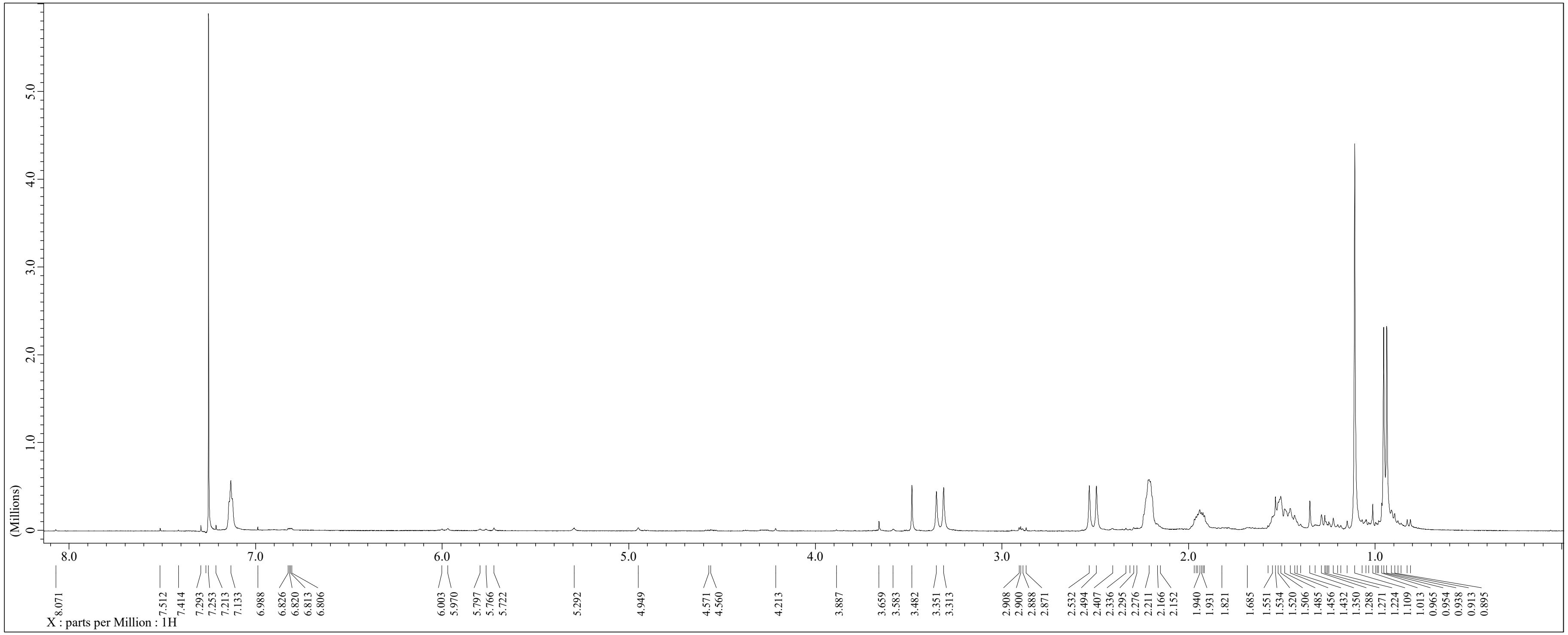

**Figure S28.** $^1$H NMR spectrum of compound **13**.

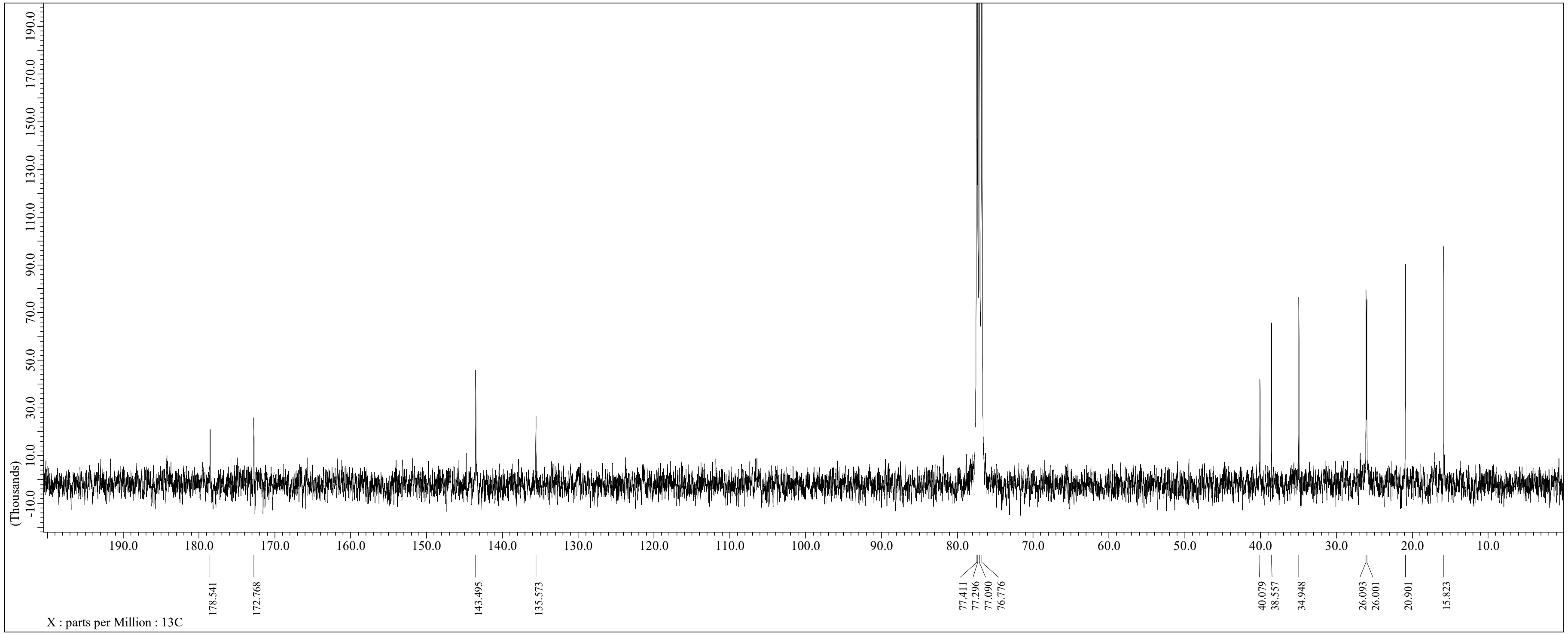


**Figure S29.** $^{13}C$ NMR spectrum of compound **13.**

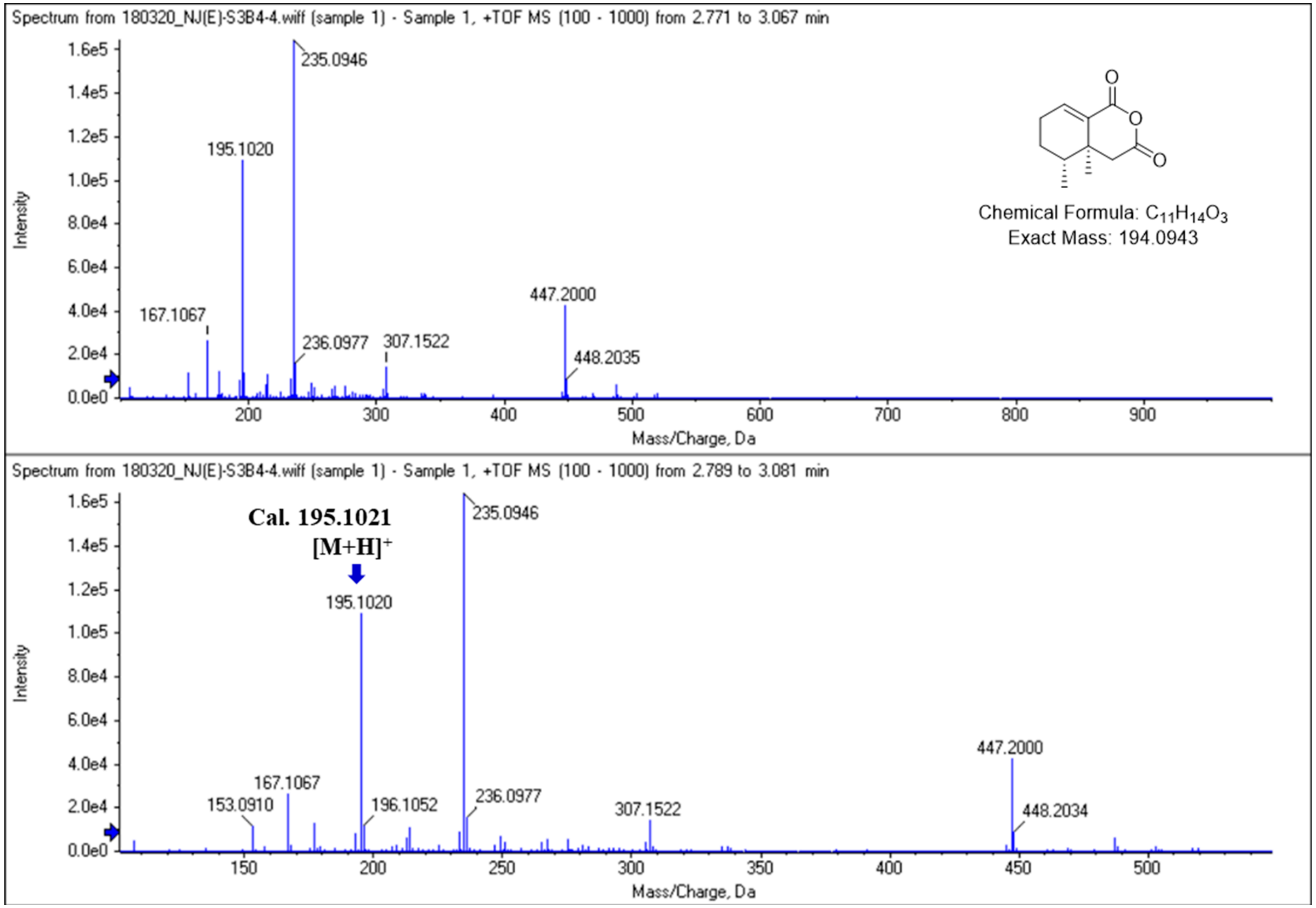


**Figure S30.** HRESI-MS spectrum of compound **13**.

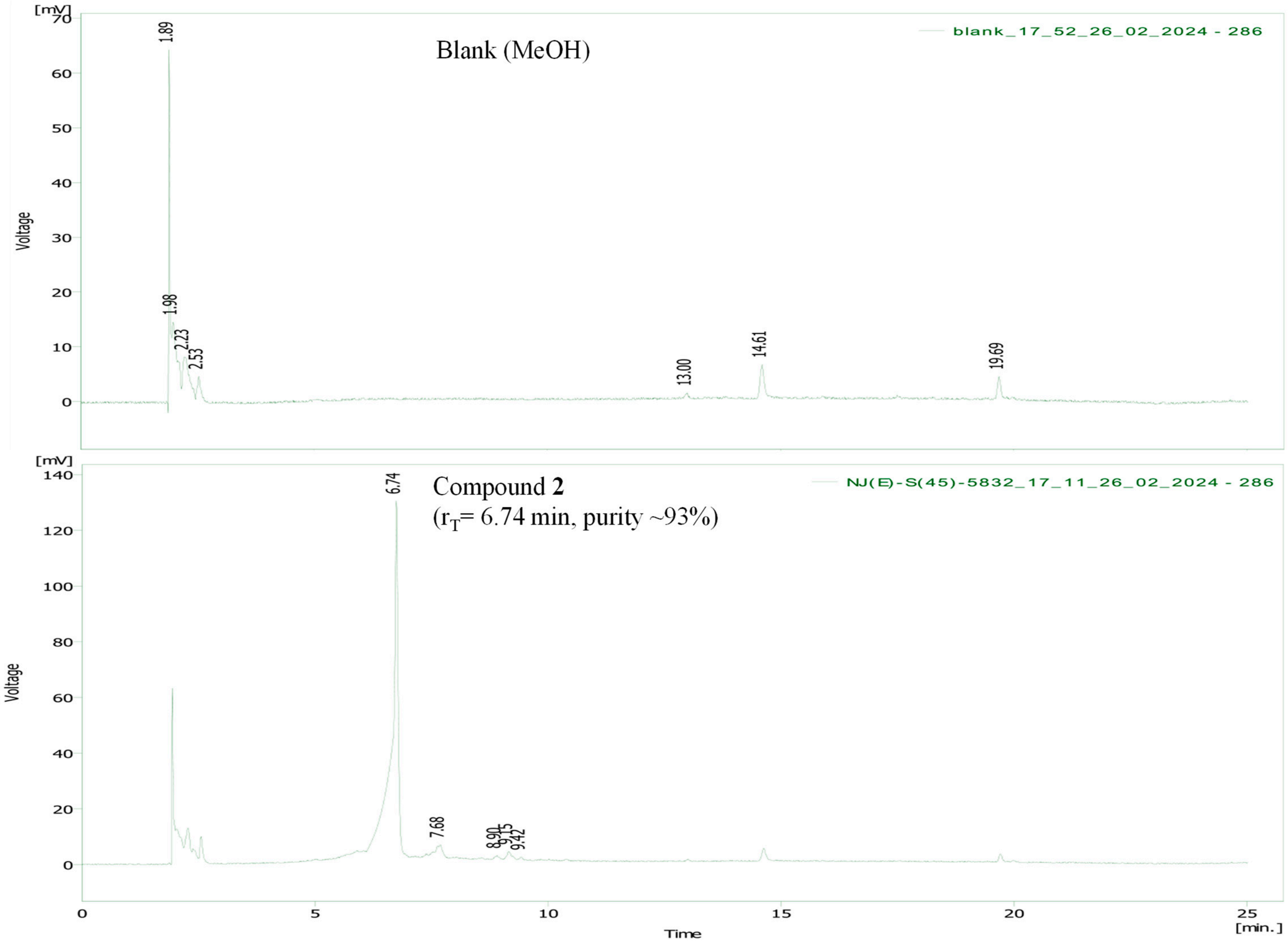


**Figure S31.** HPLC chromatogram of compound **2**.

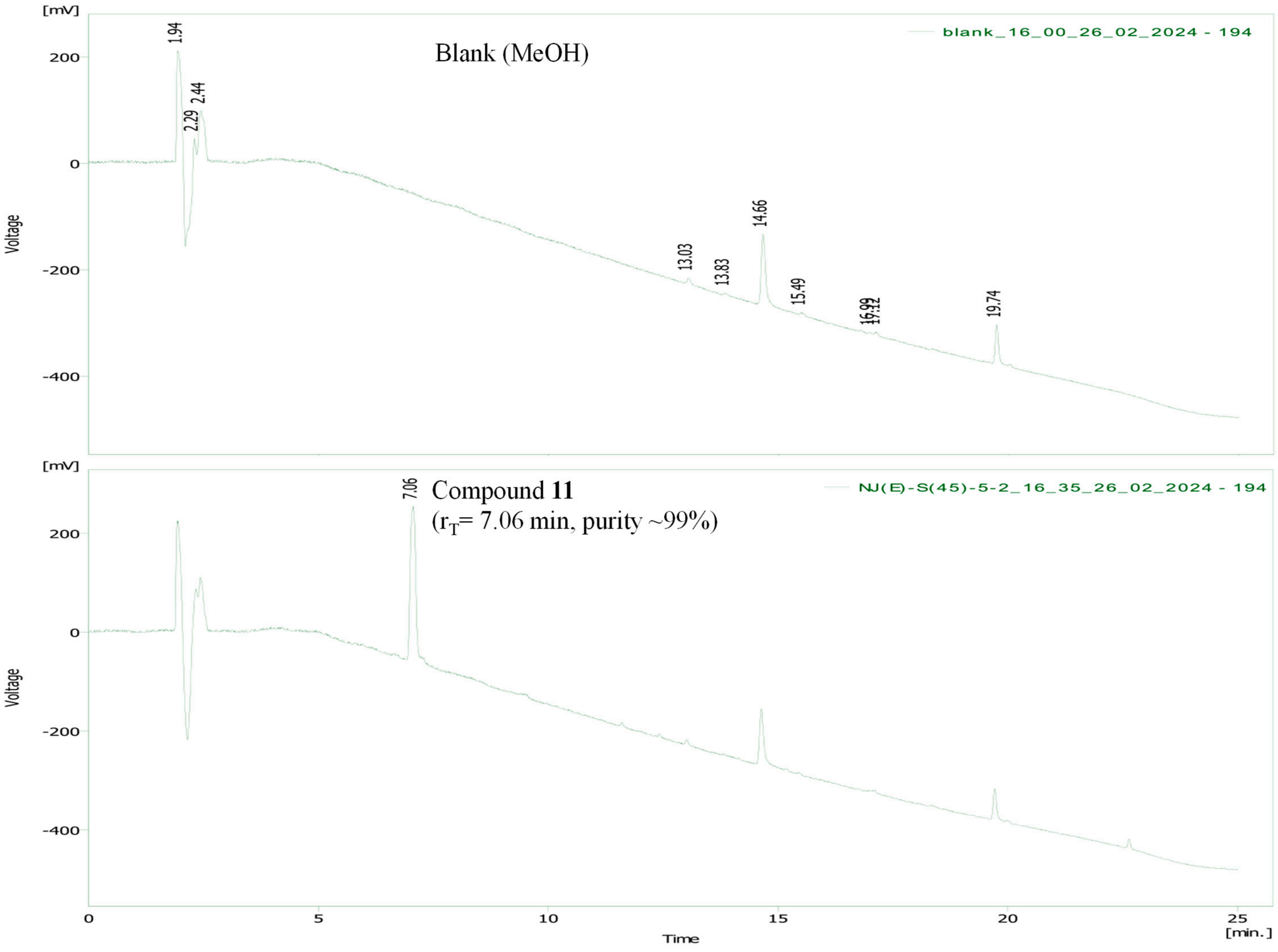


**Figure S32.** HPLC chromatogram of compound **11**.
